# Performance Analysis of Conventional Machine Learning Algorithms for Identification of Chronic Kidney Disease in Type 1 Diabetes Mellitus Patients

**DOI:** 10.3390/diagnostics11122267

**Published:** 2021-12-03

**Authors:** Nakib Hayat Chowdhury, Mamun Bin Ibne Reaz, Fahmida Haque, Shamim Ahmad, Sawal Hamid Md Ali, Ahmad Ashrif A Bakar, Mohammad Arif Sobhan Bhuiyan

**Affiliations:** 1 Department of Electrical, Electronic and Systems Engineering, Universiti Kebangsaan Malaysia, Bangi 43600, Selangor, Malaysia; nakib@baust.edu.bd (N.H.C.); mamun@ukm.edu.my (M.B.I.R.); fahmida32@yahoo.com (F.H.); sawal@ukm.edu.my (S.H.M.A.); ashrif@ukm.edu.my (A.A.A.B.); 2 Department of Computer Science and Engineering, Bangladesh Army University of Science and Technology (BAUST), Saidpur Cantonment, Saidpur 5310, Bangladesh; 3 Department of Computer Science and Engineering, University of Rajshahi, Rajshahi 6205, Bangladesh; shamim_cst@yahoo.com; 4 Department of Electrical and Electronics Engineering, Xiamen University Malaysia, Bandar Sunsuria, Sepang 43900, Selangor, Malaysia

**Keywords:** chronic kidney disease, prediction model, machine learning, type 1 diabetes mellitus

## Abstract

Chronic kidney disease (CKD) is one of the severe side effects of type 1 diabetes mellitus (T1DM). However, the detection and diagnosis of CKD are often delayed because of its asymptomatic nature. In addition, patients often tend to bypass the traditional urine protein (urinary albumin)-based CKD detection test. Even though disease detection using machine learning (ML) is a well-established field of study, it is rarely used to diagnose CKD in T1DM patients. This research aimed to employ and evaluate several ML algorithms to develop models to quickly predict CKD in patients with T1DM using easily available routine checkup data. This study analyzed 16 years of data of 1375 T1DM patients, obtained from the Epidemiology of Diabetes Interventions and Complications (EDIC) clinical trials directed by the National Institute of Diabetes, Digestive, and Kidney Diseases, USA. Three data imputation techniques (RF, KNN, and MICE) and the SMOTETomek resampling technique were used to preprocess the primary dataset. Ten ML algorithms including logistic regression (LR), k-nearest neighbor (KNN), Gaussian naïve Bayes (GNB), support vector machine (SVM), stochastic gradient descent (SGD), decision tree (DT), gradient boosting (GB), random forest (RF), extreme gradient boosting (XGB), and light gradient-boosted machine (LightGBM) were applied to developed prediction models. Each model included 19 demographic, medical history, behavioral, and biochemical features, and every feature’s effect was ranked using three feature ranking techniques (XGB, RF, and Extra Tree). Lastly, each model’s ROC, sensitivity (recall), specificity, accuracy, precision, and F-1 score were estimated to find the best-performing model. The RF classifier model exhibited the best performance with 0.96 (±0.01) accuracy, 0.98 (±0.01) sensitivity, and 0.93 (±0.02) specificity. LightGBM performed second best and was quite close to RF with 0.95 (±0.06) accuracy. In addition to these two models, KNN, SVM, DT, GB, and XGB models also achieved more than 90% accuracy.

## 1. Introduction

Diabetes mellitus (DM) is currently one of the most severe health issues facing the world, and it affects around 463 million individuals worldwide [1]. DM is considered one of the most prevalent endocrine and metabolic disorders, causing substantial damage to various organs, including the kidney [2,3]. As a result, persons with diabetes mellitus are more likely to develop chronic renal disease. According to the International Diabetes Federation (IDF), around 10% of DM patients have Type 1 diabetes mellitus (T1DM). In the T1DM population, the lifetime risk of kidney impairment is estimated to be 50% and could be as high as 70% [4]. According to the 2016 Annual Data Report of the US Renal Data System, diabetic kidney disease is one of the leading causes of end-stage renal disease (ESRD) in North America [5]. However, although ESKD has stabilized or declined in patients with type 1 diabetes over the past decades [6,7], most likely due to the increased use of renin–angiotensin system (RAS) blockers [8], it remains a life-threatening complication. Chronic kidney disease (CKD) is linked to higher morbidity and mortality in T1DM patients, and ESRD significantly increases mortality [9].

The presence of a chronic decline in renal function and structural kidney damage is diagnosed as CKD [10]. Glomerular filtration rate (GFR), which represents the amount of fluid our kidney filters per unit time, is the most precise indicator of overall kidney function [11]. Normal renal function can be defined using estimated glomerular filtration rate (eGFR), and this definition is age-dependent. An eGFR of more than 90 mL/min/1.73 m^2^ is considered as normal renal function. Although the eGFR value decreases with age, an eGFR value lower than 90 mL/min/1.73 m^2^ indicates that the kidney is not working properly. Although CKD diagnosis and classification have changed over time, according to KDIGO 2012 and current international standards, a person with an eGFR less than 60 mL/min/1.73 m^2^ for more than 3 months is considered a CKD patient [12]. Weariness, fluid retention, abnormalities in the urine, limb edema, nausea, vomiting, and neurological and cognitive impairment are the symptoms of CKD, although it can be asymptomatic in many cases [13]. Thus, there is typically a chance of a delay in recognizing, diagnosing, and treating the many etiologies of CKD, since people can be asymptomatic and need a specific laboratory-based test to identify CKD.

Furthermore, in the conventional urine protein (urinary albumin)-based CKD diagnosis technique, 24 h urine collection specimen analysis is considered the gold standard. Although the urinary albumin-to-creatinine ratio (uACR) and urinary protein-to-creatinine ratio (uPCR) currently represent excellent alternatives to the gold-standard analysis of a 24 h urine collection [14], there is still a tendency to bypass the urine albumin test. According to Medicare (a national health insurance program in the USA) claims data for diabetic patients, only half of these patients conduct tests for urine albumin [5]. However, early detection of CKD can benefit patients in receiving effective treatment because there are therapy options for slowing the progression of renal disease [15]. As CKD is ubiquitous in patients with T1DM and can be asymptomatic, an accurate prediction model that operates on easily available features can be helpful to recognize patients at higher risk of kidney function decline who may benefit from more intensive management.

The use of machine learning (ML) algorithms in addressing various disease classification problems has recently expanded due to remarkable advancements in related technologies [16,17,18]. Although there are some examples of using ML tools in kidney disease prediction [13,19,20,21,22], their use in developing CKD prediction models for type 1 diabetes mellitus patients is scarce. For example, Segal et al. [13] employed a gradient boosting tree algorithm (extreme gradient boosting implementation) to construct a model for predicting ESRD. Another study [20] established and compared nine ML models to estimate the 24 h urinary protein result response to detect CKD. These two studies did not emphasize diabetic patients.

On the other hand, Makino et al. [22] used artificial intelligence (AI) to design a prediction model for diabetic kidney diseases (DKD) based on electronic medical records (EMRs) with a 0.74 AUC score at maximum. Another research conducted by Dagliati et al. [19] applied four machine learning methods to create prediction models to identify complications of type 2 diabetes mellitus (T2DM) and achieved an accuracy of up to 0.838. Low et al. [23] used stepwise multivariable logistic regression to design a CKD progression prediction model for T2DM, where sensitivity and specificity were 75.6% and 72.3%. Some other studies also developed kidney disease prediction models in T2DM patients [24,25].

Outside some common factors, type 1 diabetes is different from type 2 diabetes [26]. Moreover, type 1 diabetes patients are diagnosed at a younger age than those with type 2 diabetes and are subjected to diabetes-related risk factors for a more extended period. Thus, adult patients with T1DM have an overall greater risk of CKD and ESKD than patients with T2DM [27,28]. However, unfortunately, limited research has been conducted to develop prediction models for CKD in T1DM patients. Vistisen et al. [8] used Poisson regression analysis to develop an ESKD prediction model in T1DM patients with C-statistics between 0.88 and 0.96. Colombo et al. [6] employed ridge regression to create a model for predicting renal disease progression in T1DM patients. To our knowledge, no other prediction models have been built to identify CKD in the type 1 diabetic population. Here, none of these models used traditional machine learning algorithms. In addition, both models included albuminuria as one of the most vital features, which is increased excretion of urinary albumin (urine protein) and a kidney damage marker. However, according to National Kidney Foundation, USA, 24 h urine collection is needed to properly detect albuminuria [29], which is inconvenient in many cases and can be overlooked easily by many asymptomatic CKD patients [5].

This study aimed to construct and compare CKD prediction models for T1DM patients utilizing 10 traditional supervised ML algorithms: logistic regression (LR), k-nearest neighbor (KNN), Gaussian naive Bayes (GNB), support vector machine (SVM), stochastic gradient descent (SGD), decision tree (DT), gradient boosting (GB), random forest (RF), extreme gradient boosting (XGB), and light gradient-boosted machine (LightGBM). Here, we included only demographic, behavioral, medical history, and biochemical blood features, which are easily available during routine follow-up of T1DM patients to predict CKD. We also applied three feature ranking techniques, random forest (RF), k-nearest neighbor (KNN), and extremely randomized trees classifier (Extra Tree), to find the relative importance of these features. In summary, this study provides a reliable machine learning-based CKD prediction model dedicated to the T1DM population. The model can operate using simple routine checkup data of T1DM patients and deliver results in no time. As a result, when a 24 h urine protein-based laboratory test is not feasible, this model can be used to predict CKD. Furthermore, all T1DM patients may utilize this model to make an educated guess on their CKD state during their regular checkups, and this will increase the likelihood of detecting asymptomatic CKD patients at an earlier stage.

## 2. Materials and Methods

### 2.1. Overall Process

**Figure 1:**
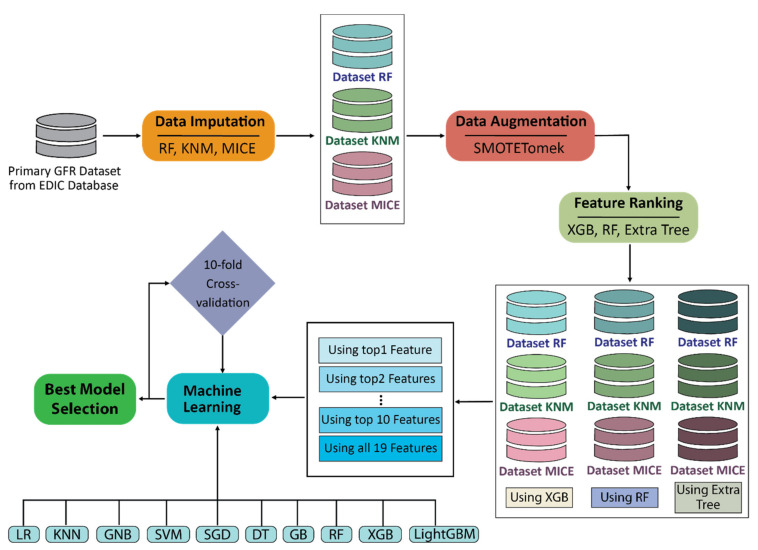
Block diagram of the overall working procedure for training and testing different machine learning models for CDK prediction in T1DM patients.

This study followed a pipeline of seven steps: primary data selection, data imputation to fill missing data, data augmentation to balance target classes, feature ranking to identify most important features, machine learning algorithms to develop models, model evaluation, and best model selection. Figure 1 illustrates the overall working procedure of training and testing different machine learning models for CDK prediction in T1DM patients.

### 2.2. Data Collection

This study used the GFR dataset from the Epidemiology of Diabetes Interventions and Complications (EDIC) clinical trial. The National Institute of Diabetes, Digestive, and Kidney Diseases (Bethesda MD, Montgomery, Maryland, USA) conducted this trial to observe the impact of intensive diabetes treatment on the T1DM population [30,31]. The EDIC study started with 1375 T1DM patients in 1994 and is still going [32]. Here 48% of patients were female, and 52% of them were male.

The EDIC study collects data at 28 EDIC clinic sites across the US and Canada; this ensured diversity in patient types. This study is a longitudinal study, whereby the patients’ initial age range was between 19 years and 57 years, and, after decades of data collection, this study had patient’ information from age 19 years to 80 years. According to the International Diabetic Federation (IFD), most T1DM patients are adults aged 20–79 years [1].

In the EDIC study, serum creatinine levels were measured annually throughout the period at the EDIC Central Biochemistry Laboratory, University of Minnesota [31], using an automated kinetic modification of the Jaffe reaction on a Beckman Synchron CX3 Clinical C System [33,34]. The Chronic Kidney Disease Epidemiology Collaboration (CKD-EPI) formula was used to calculate estimated GFR (eGFR) using data on serum creatinine levels, age, sex, and race [31,35]. A sustained eGFR value <60 mL/min/1.73 m^2^ on at least two consecutive collections was considered an abnormal eGFR.

During the EDIC trial, participants’ body mass index (BMI), blood pressure (BP), and glycated hemoglobin (HbA1c) levels were all measured yearly [36]. The presence of systolic BP ≥ 140 and/or diastolic BP ≥90 mmHg on two successive yearly visits was considered as incident hypertension. Pulse pressure (PP) was calculated using the difference between systolic and diastolic pressure [36]. The albumin excretion rate (AER) and fasting lipid levels (cholesterol, triglycerides, HDL, and LDL) were measured every 2 years [31]. That study also included other physical (sex, age, weight), behavioral (smoking, drinking), medication use (use of antihypertensive medications, ACE inhibitors, and lipid-lowering agents), and diabetes-specific (daily insulin dose and duration of diabetes) information [36].

Demographic and behavioral data were assessed by self-report, whereas experienced persons checked blood pressure, and medication use was assessed yearly by self-report [35]. All laboratory measurements were carried out using standardized methods in the EDIC central biochemistry laboratory, and long-term quality control mechanisms were in place to guard against measurement drift [36,37].

In our research, we considered data of 1375 participants over 16 years of the EDIC study from 1994 to 2010. After removing all duplicate data, we finally selected 3184 samples in total. In our dataset, the target variable had two classes: CKD represented as 1 and non-CKD represented as 0. We used an eGFR value of less than 60 mL/min/1.73 m^2^ on at least two consecutive collections to define CKD as defined by KDIGO 2012 [12]. Furthermore, other studies utilized the same measurement to indicate substantial GFR deterioration [33,36,37,38,39,40], and it is considered evidence of CKD [10,11].

In our study, along with medical history, demographics, and behavioral information, we only considered laboratory data available through routine checkups of a T1DM patient. In total, we included 19 parameters: age, sex, BMI, smoking and drinking habit, hypertension, use of ACE inhibitors and antihypertensive medicine, daily insulin dose, hypercholesterolemia, duration of insulin-dependent diabetes mellitus (IDDM), glycated hemoglobin (HbA1c) levels, total cholesterol, triglycerides, high-density lipoproteins (HDL), low-density lipoproteins (LDL), systolic blood pressure (SBP), diastolic blood pressure (DBP), and mean blood pressure. These parameters were considered essential for CKD detection in other studies [13,19,20,38,39,40]. To avoid overfitting problems, we did not consider parameters such as albumin excretion rate (AER), serum creatinine, and current GFR because serum creatinine is used to calculate eGFR, and AER is a CKD identifier [10,24,35]. Moreover, 24 h urine is necessary to calculate albumin [29], whereas this study aimed to develop a prediction model which would quickly predict CDK with easily available routine checkup data.

### 2.3. Data Imputation

**Figure 2:**
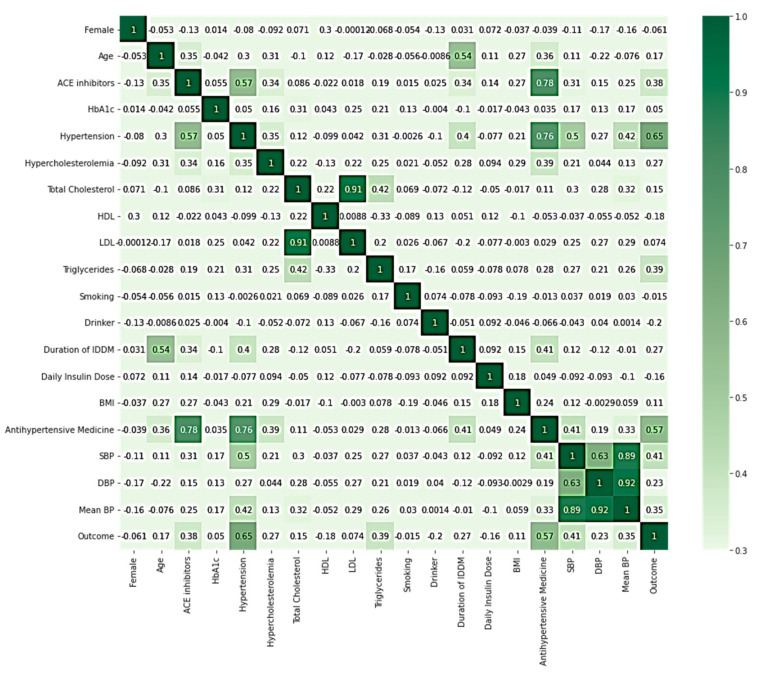
Features correlation heatmap of Dataset KNN.

Our primary dataset had 3184 samples, with 68 missing values in five features: sex, smoking and drinking habit, use of ACE inhibitors, and daily insulin dose. We used three data imputation techniques, random forest (RF), k-nearest neighbors (KNN), and multiple imputation by chained equations (MICE), to fill missing values [41,42,43]. Thus, we created three datasets using three different imputation methods: Dataset RF, Dataset KNN, and Dataset MICE. Figure 2 represents the correlation heatmap of Dataset KNN. Other datasets also provided similar correlation heatmaps (Supplementary Figures S1 and S2).

### 2.4. Data Augmentation

Our datasets were imbalanced; a total of 391 of 3184 subjects had CKD. Therefore, we used the SMOTETomek technique, which combines the synthetic minority oversampling technique (SMOTE) and the Tomek links undersampling techniques to balance the dataset [44,45].

### 2.5. Feature Ranking

To find feature importance, we applied three feature ranking models to each dataset. We developed these models using extreme gradient boosting (XGB), random forest (RF), and extremely randomized trees classifier (Extra Tree) [41,46,47]. Then, we rearranged all 19 features on the basis of their relative importance. Thus, in combination, we had three datasets with three feature rankings for each dataset.

### 2.6. ML Model Development

This research examined the performance of conventional ML algorithm-based CKD classifiers on T1DM patients. Here, we applied 10 traditional supervised ML algorithms: logistic regression (LR), k-nearest neighbor (KNN), Gaussian naïve Bayes (GNB), support vector machine (SVM), stochastic gradient descent (SGD), decision tree (DT), gradient boosting (GB), random forest (RF), extreme gradient boosting (XGB), and light gradient-boosted machine (LightGBM) to develop 10 different prediction models. We used all three datasets with three different feature ranking strategies for each model to find out the best combination of data imputation technique, feature ranking technique, and number of features.

In-house-built Python 3.7 codes using the Scikit-learn machine learning library [48] were applied to develop all ML models for prediction, data imputation, augmentation, and feature ranking. To train and test the developed ML models, we used stratified k-fold cross-validation where the value of k was 10. In this work, a multiclass SVM model was considered. The KNN model was created for 25 nearest neighbors, and the RF model used a 100-bagged decision tree.

### 2.7. Statistical Analysis

All statistical analyses for baseline EDIC patient characteristics were performed contrasting the CKD and Non-CKD groups. Our data had both continuous and categorical parameters. We calculated the mean ± standard deviation (SD), standard error of the mean (SEM), maximum and minimum value, 95% confidence interval, and correlation for continuous features. An independent *t*-test was used to find out the 95% confidence intervals, and the correlation between different variables and CKD was evaluated using Pearson’s correlation coefficient with *p*-values. Table 1 shows the baseline factors of continuous features of EDIC patients to better understand the patients’ characteristics.

On the other hand, Table 2 presents the baseline characteristics of categorical parameters. Here, all categorical features had binary values (0 or 1). We used the same method to calculate the correlation coefficient. In-house build Python 3.7 codes were applied to perform all statistical analyses.

## 3. Results

### 3.1. Preparing Datasets

In the first year, the average age of EDIC patients was 35.093 (±6.98) years, with a mean diabetes duration of 13.64 (±4.94) years. Initially, we had only four patients with CKD. However, across the 16 years of EDIC study, another 66 patients got CKD. We considered data over the 16 years of the EDIC study, 20,394 samples in total, and finally selected 3184 participants, with 391 of them having CKD. After processing the primary dataset using three data imputations and the SMOTETomek augmentation technique, each of the final three datasets was prepared with 2790 (±10) samples per class.

**Figure 3:**
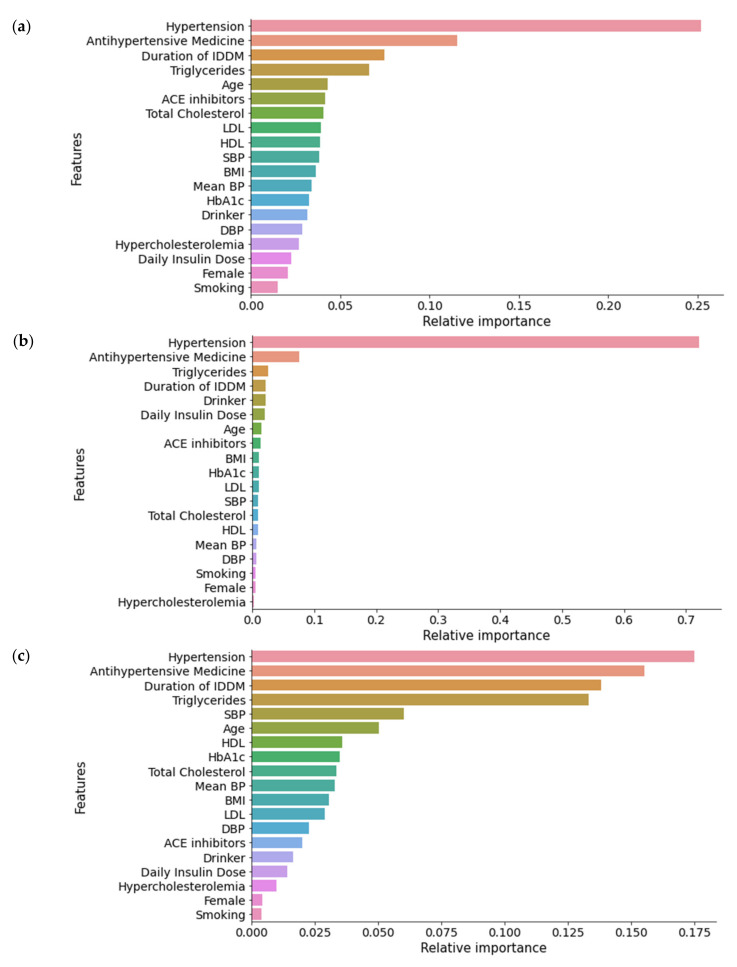
Feature ranking on Dataset KNN: (**a**) Extra Tree algorithm; (**b**) XGB algorithm; (**c**) RF algorithm.

We applied three different feature ranking algorithms (XGB, RF, Extra Tree) on augmented datasets to create three separate feature rankings for each dataset. Here, we found hypertension, antihypertensive medication, and duration of IDDM as the three most important features in most cases. All three algorithms returned hypertension as the most important feature in all datasets. Other significant characteristics were triglycerides, ACE inhibitors, age, SBP, HDL, LDL, total cholesterol, drinking, mean BP, BMI, drinker, daily insulin dose, and HbA1c. However, the position of different features and their relative importance value varied significantly in these lists. Figure 3 represents the relative importance of features using the Extra Tree, XGB, and RF techniques on Dataset KNN. We observed that each feature ranking model returned almost identical results on every dataset (Dataset RF, Dataset KNN, and Dataset MICE); details can be found in the Supplementary Materials (Supplementary Figures S3–S5).

### 3.2. Performance Evaluation of ML Models

After applying three different data imputation techniques, we had three different datasets (Dataset RF, Dataset KNN, and Dataset MICE), and we applied three different feature ranking methods (XGB, RF, Extra Tree) to these three datasets. In total, we had nine combinations of different data imputation techniques and feature ranking models, and we implemented different machine learning algorithms to construct CKD prediction models using all these combinations. For each combination, we trained and evaluated every ML model using the top feature, then the top two features, the top three features, etc., continuing for all 19 features, to identify the best combination of feature ranking model, data imputation technique, and minimum number of features to achieve the best performance. We applied 10 conventional ML algorithms, LR, KNN, GNB, SVM, SGD, DT, GB, RF, XGB, and LightGBM, to develop CKD prediction models and used 10-fold stratified cross-validation to train and test every model, with a 9:1 training/test data ratio.

No significant difference was found in performance using different data imputation techniques. We had very few missing values, which could explain why alternative data imputation techniques had a minimal effect on model performance. On the other hand, the minimum number of features required to achieve the optimal model performance varied significantly between feature ranking techniques.

**Figure 4:**
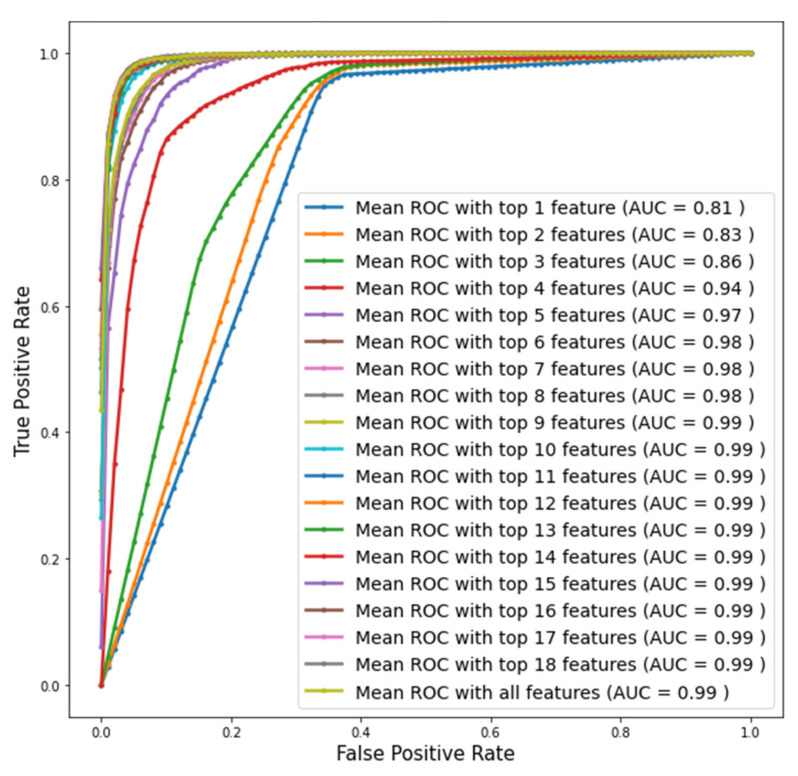
ROC curve of RF model with different features ranked by XGB.

The RF classifier model achieved the highest result with the XGB feature ranking method. This model used 11 features to reach 0.96 (±0.01) accuracy with 0.98 (±0.01) sensitivity and 0.93 (±0.02) specificity. For this model, selected variables were hypertension, antihypertensive medicine, triglycerides, duration of IDDM, drinker, daily insulin dose, age, ACE inhibitors, BMI, HbA1c, and LDL. Figure 4 shows the ROC curve of the RF model using 1–19 features ranked by the XGB feature ranking technique on Dataset KNN. The ROC curves for the best models of the other algorithms can be found in the Supplementary Materials (Supplementary Figures S6–S14).

LightGBM came in second place and was pretty close to RF in terms of accuracy, with 0.95 (±0.06). In addition to these two models, KNN, SVM, DT, GB, and XGB models obtained greater than 90% accuracy. Despite having a lower sensitivity than several algorithms, SVM had the best specificity. The performance of the best models for each of the 10 ML algorithms employing three feature ranking approaches is shown in Table 3. The overall best model is shaded. Details of these models can be found in the Supplementary Materials (Supplementary Tables S1–S10). In Table 3, we only considered the KNN data imputation technique. The outcomes of the other two data imputation approaches were nearly identical.

## 4. Discussion

At present, one of the fastest-growing diseases is diabetes mellitus (DM), with approximately 463 million people suffering worldwide [1]. DM patients have a higher risk of developing serious health problems that can affect the heart, eyes, kidneys, nerves, and teeth. According to the International Diabetes Federation (IDF), the leading cause of kidney failure in developed countries is diabetes [3]. Furthermore, IDF estimates that around 10% of these DM patients suffer from type 1 diabetes mellitus (T1DM). Although T1DM can affect people at any age, it generally develops among young adults. As a result, they are exposed to diabetes-related risk factors for a more extended period. Chronic kidney disease (CKD) is one of the most significant complications of T1DM, and about half of the patients with T1DM have a lifetime risk of developing CKD [4].

The most important accessible indicator of overall kidney function is the glomerular filtration rate (GFR). It represents the amount of fluid filtered through the kidney per unit of time [11]. The estimated glomerular filtration rate (eGFR) can be used to define the normal renal function, and this definition is age-dependent. The eGFR value decreases with age, but it needs to be greater than 90 mL/min/1.73 m^2^ to be considered normal renal function, whereas people with eGFR less than 60 mL/min/1.73 m^2^ for more than 3 months are considered CKD patients [12,15].

Moreover, CKD is hard to detect, as it can be asymptomatic in many cases. People with CKD for a long period may not exhibit any symptoms, and, because of this asymptomatic nature, there is a typical chance of delay in its recognition [13]. Furthermore, there is a tendency to bypass the traditional urine protein (urinary albumin)-based CKD diagnostic approach. According to Medicare (a national health insurance program in the United States), barely half of the diabetes individuals get a urine albumin test [5]. Early detection of CKD can be helpful to prevent the risk of end-stage kidney disease (ESKD) through intensive management. As T1DM patients have an enormous risk of developing CKD, a prediction model that can predict CKD from patients’ routine checkup data would greatly help them.

Machine learning (ML) approaches are now being explored in various medical systems. Due to the recent boost in related technology, applying ML techniques has become easier. Health professionals are more enthusiastic about using their advantages of flexibility and self-learning capacity as an aiding system for reliable performance. Intelligent systems based on ML algorithms have been intensively investigated for various biomedical systems, focusing on disease detection and risk reduction [16,17,18,49,50]. Like other severe diseases, CKD has piqued the interest of researchers in creating ML-based diagnosis systems for CKD [3,13,18,20,21,22]. However, their application in developing prediction models for CKD in type 1 diabetic mellitus patients is rare.

In the literature, several ML-based kidney disease classifier models have been reported; however, most of them did not focus on diabetes mellitus patients. For example, Segal et al. [13] used the extreme gradient boosting algorithm to build a prediction model to identify end-stage renal disease (ESRD) progression for patients who already have CKD. In addition, Xiao et al. [20] targeted predicting 24 h urinary protein outcomes to detect CKD by applying different ML models. They included logistic regression, Elastic Net, lasso regression, ridge regression, support vector machine, random forest, XGBoost, neural network, and k-nearest neighbor and got the highest AUC of 0.873.

Some studies only considered type 2 diabetes mellitus (T2MD) patients to develop kidney disease prediction models. Low et al. [23] applied multivariable logistic regression to design a CKD progression prediction model in patients with T2DM, where both sensitivity and specificity were below 80%. In another study, Dunkler et al. [25] designed a multinomial logistic model to predict CKD risk in individuals with type 2 diabetes.

Although type 1 diabetes and type 2 diabetes share some common characteristics, they are different [26]. Nevertheless, T1DM occurs at a younger age and stays for a longer period than T2DM. As a result, T1DM patients have a greater risk of kidney diseases (including CKD) than T2DM patients. Thus, a CKD prediction model solely concentrating on T1DM patients would be more appropriate. Unfortunately, we found only two studies focused on developing kidney disease prediction models for T1DM patients.

Vistisen et al. [8] developed and evaluated an ESKD prediction model in T1DM patients using Poisson regression analysis and achieved C-statistic values between 0.88 and 0.96. Their study used albuminuria, smoking status, physical activity, alcohol intake, antihypertensive treatment, lipid-lowering treatment, RAS-blocker treatment, eGFR, and previous cardiovascular disease as variables and achieved a C-statistic of 0.888 (95% CI 0.849–0.927) in the derivation cohort. In another study conducted by Colombo et al. [6], ridge regression was implemented to build a model to predict renal diseases in T1DM patients. That study included serum creatinine, urinary albumin/creatinine ratio (ACR), age, sex, diabetes duration, follow-up time, HbA1c, and prior cardiovascular disease information to predict final eGFR with an *r^2^* of 0.745 (*p* < 10^−16^). Both studies used albuminuria as a parameter, which designates increased excretion of one kind of urine protein (urinary albumin). Albuminuria is generally used as a marker of kidney damage, but its measurement is lengthy. National Kidney Foundation, USA recommends using 24 h urine to measure albuminuria [29], which is not convenient for many patients, and barely half of the USA diabetes patients get this urine protein-based test [5]. Asymptomatic CKD patients in particular can exclude this test from their routine checkup due to this inconvenience. Moreover, traditional machine learning algorithms were not considered in these two studies.

This study applied and evaluated 10 traditional machine learning algorithms to build prediction models to quickly predict CKD in T1DM patients from easily available routine follow-up data. We used 16 years of data of 1375 type 1 diabetes mellitus patients from the clinical trials of the Epidemiology of Diabetes Interventions and Complications (EDIC) [30,31]. Our study included age, sex, BMI, smoking and drinking habit, hypertension, hypercholesterolemia, duration of insulin-dependent diabetes mellitus (IDDM), use of ACE inhibitors and antihypertensive medicine, daily insulin dose, glycated hemoglobin (HbA1c) levels, total cholesterol, triglycerides, high-density lipoproteins (HDL), low-density lipoproteins (LDL), systolic blood pressure (SBP), diastolic blood pressure (DBP), and mean blood pressure. These parameter values are easily available through routine checkups of a T1DM patient and have been considered in other clinical models for predicting renal function decline in diabetes patients [13,19,20,38,39,40]. We used the KDIGO 2012 [12] definition of CKD; an eGFR value of less than 60 mL/min/1.73 m^2^ for more than three months was considered as CKD. We did not include serum creatinine, albumin excretion rate (AER), and current GFR to avoid overfitting, as serum creatinine is the most important parameter to calculate eGFR and AER is itself a CKD identifier. In addition, 24 h urine analysis is necessary to measure AER.

Our data had missing values and class imbalance. Three AI-based data imputation techniques, random forest (RF), k-nearest neighbors (KNN), and multiple imputation by chained equations (MICE), were used to fill missing values [41,42,43]. In addition, we used a combination of oversampling and undersampling techniques SMOTETomek to address class imbalance [51]. We used extreme gradient Boosting (XGB), random forest (RF), and extremely randomized trees classifier (Extra Tree) for feature ranking and to select the 10 most significant features [41,46,47]. Thus, we had nine distinct combinations of different data imputation approaches and feature ranking models. We used 10 machine learning algorithms, logistic regression (LR), k-nearest neighbor (KNN), Gaussian naive Bayes (GNB), support vector machine (SVM), stochastic gradient descent (SGD), decision tree (DT), gradient boosting (GB), random forest (RF), extreme gradient boosting (XGB), and light gradient-boosted machine (LightGBM), to develop different prediction models for classifying CKD utilizing all nine combinations. We trained and assessed each ML model using the top feature, then the top two features, the top three features, and so on, until we found the optimum combination of feature ranking model, data imputation technique, and number of features to achieve the best performance. We employed 10-fold stratified cross-validation to evaluate different models, with a 9:1 training/test data ratio.

Hypertension, antihypertension medicine, and duration IDDM were the top three features in most feature ranking techniques. Triglycerides, ACE inhibitors, age, SBP, HDL, LDL, total cholesterol, drinking, mean BP, BMI, drinker, daily insulin dose, and HbA1c were other top features, but their positions and relative importance values were different for different models. We had few missing values, and different data imputation techniques showed no significant difference in performance.

With the XGB feature ranking technique and top 11 features, the RF classifier algorithm produced the best CKD prediction model with 0.96 (±0.01) accuracy, 0.98 (±0.01) sensitivity, and 0.93 (±0.02) specificity. LightGBM came in second with 0.95 (±0.06) accuracy. In addition to these two models, the accuracy of KNN, SVM, DT, GB, and XGB models was more than 90%. SVM had the greatest specificity while having a lower sensitivity than several algorithms.

In this study, conventional machine learning algorithms were used to develop a CKD prediction model in T1DM patients for the first time. Here, the suggested model showed reliable performance with more than 95% accuracy. Moreover, to operate this model, we do not need to collect 24 h urine protein or other critical values. Only general data from routine follow-up of a T1DM patient is enough to produce an accurate result without any delay. Consequently, this model can be used to predict CKD when critical laboratory tests are not possible. In addition, all T1DM patients may use this model to make an educated prediction of their CKD status during a regular checkup, and this can improve the chances of discovering asymptomatic CKD patients at an earlier stage.

## 5. Conclusions

CKD is one of the most common diabetes-related complexities, and almost 50% of T1DM patients have a lifetime risk. Diagnosis of CKD is complicated because it can be asymptotic even in the late stages. Although there are some prediction models to detect CKD in T2DM patients, this is a rare approach in T1DM patients, and none of them use traditional ML algorithms. Nevertheless, the application of ML in several biomedical fields has shown a positive influence on enhancing performance over conventional methods. This study investigated the performance of various common ML approaches (LR, KNN, GNB, SVM, SGD, DT, GB, RF, XGB, and LightGBM) in the diagnosis and stratification of CKD in T1DM patients. We used general features available from a routine checkup. This analysis found that the models developed by the random forest (RF) algorithm with all 19 variables worked better in CKD classification. Therefore, a random forest or LightGBM-based CKD prediction technique can help healthcare professionals to identify potential CKD patients in T1DM patients and refer them for further investigation.

## Figures and Tables

**Table 1:** Baseline Characteristics of The EDIC Patients (Continuous Features).

N = 1375	Mean	SEM	Min	Max	95% Confidence Interval		Pearson Correlation	
					Lower Limit	Upper Limit	*r*	*p*
Age (years)	35.093 ± 6.98	0.18	19.00	57.00	34.72	35.45	0.039	0.13
BMI (kg/m^2^)	26.09 ± 4.04	0.11	16.62	66.01	25.88	26.30	−0.02	0.33
Diabetic duration (years)	13.64 ± 4.94	0.13	6.00	28.00	13.38	13.90	0.08	<0.05
Hba1c (%)	8.14 ± 1.39	0.03	4.40	15.10	8.07	8.22	0.03	0.25
HDL cholesterol (mg/dL)	52.50 ± 13.06	0.35	25.00	103.00	51.81	53.18	−0.03	0.18
LDL cholesterol (mg/dL)	114.02 ± 30.52	0.81	26.00	310.00	112.42	115.62	0.03	0.14
Total cholesterol (mg/dL)	183.71 ± 35.87	0.96	85.00	444.00	181.83	185.59	0.04	0.11
Triglycerides (mg/dL)	86.79 ± 64.30	1.72	17.00	1110.00	83.42	90.16	0.05	<0.05
Systolic BP (mm Hg)	117.35 ± 12.61	0.33	82.00	172.00	116.69	118.01	0.07	<0.05
Diastolic BP (mm Hg)	74.99 ± 9.27	0.25	40.00	116.00	74.50	75.47	0.04	0.07
Mean BP (mm Hg)	89.11 ± 9.36	0.25	59.33	134.00	88.62	89.60	0.06	<0.05

**Table 2:** Baseline characteristics of EDIC patients (categorical features).

N = 1375	Number of Positive Outcomes	Number of Negative Outcomes	Pearson Correlation	
			*r*	*p*
Female	659	716	−0.01	0.47
ACE inhibitors	87	1288	0.20	1.44
Hypertension	228	1147	0.11	1.10
Hypercholesterolemia	402	973	0.03	0.26
Smoking	273	1102	−0.01	0.84
Drinking	486	889	−0.02	0.33
Daily insulin dose	255	1120	0.00	0.90
Antihypertensive medicine	128	1247	0.16	5.04

**Table 3:** Comparative performance analysis of different ML models.

Algorithm	Data Imputation	Feature Selection Models	Number of Features	Sensitivity (Recall)	Specificity	Accuracy	Precision	F1_Score	Non-CKD		CKD	
									True Negative	False Positive	False Negative	True Positive
LR	KNN	XGB	11	0.90 (±0.02)	0.76 (±0.04)	0.83 (±0.01)	0.79 (±0.03)	0.84 (±0.01)	2124	657	290	2500
		RF	16	0.89 (±0.03)	0.76 (±0.05)	0.83 (±0.01)	0.79 (±0.03)	0.84 (±0.01)	2115	666	300	2490
		Extra Tree	8	0.93 (±0.04)	0.72 (±0.04)	0.83 (±0.01)	0.77 (±0.02)	0.84 (±0.01)	1999	782	187	2603
KNN	KNN	XGB	17	0.99 (±0.01)	0.80 (±0.04)	0.90 (±0.02)	0.83 (±0.03)	0.91 (±0.01)	2229	553	24	2766
		RF	9	0.99 (±0.01)	0.81 (±0.03)	0.90 (±0.02)	0.84 (±0.02)	0.91 (±0.01)	2242	540	32	2758
		Extra Tree	9	0.99 (±0.02)	0.81 (±0.03)	0.90 (±0.01)	0.84 (±0.02)	0.91 (±0.01)	2240	542	33	2757
GNB	KNN	XGB	15	0.93 (±0.03)	0.75 (±0.05)	0.84 (±0.02)	0.79 (±0.03)	0.85 (±0.01)	2074	708	203	2587
		RF	16	0.91 (±0.03)	0.75 (±0.04)	0.83 (±0.01)	0.78 (±0.02)	0.84 (±0.01)	2083	699	264	2526
		Extra Tree	17	0.90 (±0.02)	0.75 (±0.04)	0.82 (±0.02)	0.78 (±0.02)	0.84 (±0.01)	2085	697	289	2501
SVM	KNN	XGB	9	0.88 (±0.06)	0.94 (±0.01)	0.91 (±0.02)	0.93 (±0.01)	0.90 (±0.03)	2603	179	335	2455
		RF	4	0.92 (±0.04)	0.82 (±0.05)	0.87 (±0.01)	0.84 (±0.03)	0.87 (±0.01)	2284	498	233	2557
		Extra Tree	5	0.92 (±0.03)	0.83 (±0.04)	0.88 (±0.01)	0.84 (±0.03)	0.88 (±0.01)	2301	481	210	2580
SGD	KNN	XGB	4	0.96 (±0.01)	0.72 (±0.28)	0.81 (±0.02)	0.74 (±0.02)	0.83 (±0.01)	1822	961	104	2686
		RF	2	0.96 (±0.01)	0.69 (±0.06)	0.80 (±0.02)	0.60 (±0.60)	0.83 (±0.03)	1892	891	205	2585
		Extra Tree	3	0.96 (±0.01)	0.65 (±0.03)	0.81 (±0.02)	0.74 (±0.02)	0.83 (±0.01)	2013	770	642	2148
DT	KNN	XGB	17	0.94 (±0.03)	0.91 (±0.01)	0.93 (±0.02)	0.91 (±0.02)	0.93 (±0.02)	2533	249	153	2637
		RF	15	0.94 (±0.05)	0.90 (±0.02)	0.92 (±0.03)	0.90 (±0.02)	0.92 (±0.03)	2504	278	170	2620
		Extra Tree	10	0.93 (±0.09)	0.91 (±0.03)	0.92 (±0.04)	0.91 (±0.03)	0.92 (±0.05)	2525	257	212	2578
GB	KNN	XGB	15	0.93 (±0.07)	0.87 (±0.02)	0.90 (±0.04)	0.87 (±0.01)	0.90 (±0.04)	2411	371	207	2583
		RF	11	0.93 (±0.06)	0.86 (±0.02)	0.90 (±0.02)	0.87 (±0.01)	0.90 (±0.03)	2403	379	198	2592
		Extra Tree	9	0.93 (±0.08)	0.86 (±0.02)	0.90 (±0.03)	0.87 (±0.02)	0.90 (±0.04)	2395	387	195	2595
RF	KNN	XGB	11	0.98 (±0.01)	0.93 (±0.01)	0.96 (±0.01)	0.94 (±0.01)	0.96 (±0.01)	2593	189	59	2731
		RF	15	0.99 (±0.01)	0.93 (±0.03)	0.96 (±0.01)	0.93 (±0.02)	0.96 (±0.01)	2585	197	40	2750
		Extra Tree	12	0.99 (±0.01)	0.93 (±0.02)	0.96 (±0.01)	0.93 (±0.02)	0.96 (±0.01)	2588	194	41	2749
XGB	KNN	XGB	13	0.95 (±0.04)	0.88 (±0.03)	0.92 (±0.02)	0.89 (±0.02)	0.92 (±0.02)	2439	343	127	2663
		RF	12	0.96 (±0.04)	0.87 (±0.02)	0.92 (±0.02)	0.88 (±0.01)	0.92 (±0.02)	2432	350	120	2670
		Extra Tree	10	0.96 (±0.03)	0.87 (±0.02)	0.92 (±0.02)	0.88 (±0.01)	0.92 (±0.02)	2409	373	98	2692
Light GBM	KNN	XGB	12	0.96 (±0.16)	0.94 (±0.04)	0.95 (±0.06)	0.95 (±0.03)	0.95 (±0.07)	2626	157	119	2671
		RF	13	0.96 (±0.16)	0.94 (±0.03)	0.95 (±0.06)	0.94 (±0.03)	0.95 (±0.07)	2617	166	125	2665
		Extra Tree	12	0.96 (±0.16)	0.94 (±0.04)	0.95 (±0.06)	0.95 (±0.03)	0.95 (±0.07)	2626	157	125	2665

## Data Availability

Restrictions apply to the availability of these data. Data were obtained from the National Institute of Diabetes and Digestive and Kidney Diseases (NIDDK) (Bethesda, MD, USA) and are available (https://repository.niddk.nih.gov/studies/edic/, accessed on 6 June 2021) with the permission of NIDDK.

## Supplementary Materials

The following are available online at https://www.mdpi.com/article/10.3390/diagnostics11122267/s1: Figure S1. Feature correlation heatmap of Dataset RF; Figure S2. Feature correlation heatmap of Dataset MICE; Figure S3. Feature ranking using Extra Tree algorithm: (a) Dataset KNN; (b) Dataset MICE; (c) Dataset RF; Figure S4. Feature ranking using RF algorithm: (a) Dataset KNN; (b) Dataset MICE; (c) Dataset RF; Figure S6. ROC curve of LR model with different features ranked by Extra Tree on Dataset KNN; Figure S7. ROC curve of KNN model with different features ranked by Extra Tree on Dataset KNN; Figure S8. ROC curve of GNB model with different features ranked by XGB on Dataset KNN; Figure S9. ROC curve of SVM model with different features ranked by XGB on Dataset KNN; Figure S10. ROC curve of SGD model with different features ranked by Extra Tree on Dataset KNN; Figure S11. ROC curve of DT model with different features ranked by XGB on Dataset KNN; Figure S12. ROC curve of GB model with different features ranked by XGB on Dataset KNN; Figure S13. ROC curve of XGB model with different features ranked by Extra Tree on Dataset KNN; Figure S14. ROC curve of LightGMB model with different features ranked by XGB on Dataset KNN; Table S1. Performance analysis of LR algorithm using KNN data imputation and Extra Tree feature ranking; Table S2. Performance analysis of KNN algorithm using KNN data imputation and Extra Tree feature ranking; Table S3. Performance analysis of GNB algorithm using KNN data imputation and XGB feature ranking; Table S4. Performance analysis of SVM algorithm using KNN data imputation and XGB feature ranking; Table S5. Performance analysis of SGD algorithm using KNN data imputation and Extra Tree feature ranking; Table S6. Performance analysis of DT algorithm using KNN data imputation and XGB feature ranking; Table S7. Performance analysis of GB algorithm using KNN data imputation and Extra Tree feature ranking; Table S8. Performance analysis of RF algorithm using KNN data imputation and XGB feature ranking; Table S9. Performance analysis of XGB algorithm using KNN data imputation and Extra Tree feature ranking; Table S10. Performance analysis of LightGBM algorithm using KNN data imputation and XGB feature ranking.

## References

[1] International Diabetes Federation “IDF Diabetes Atlas Ninth.” Dunia: IDF (2019). https://www.idf.org/e-library/welcome.html.

[2] Haque F., Reaz M.B.I., Chowdhury M., Srivastava G., Ali S.H.M., Bakar A., Bhuiyan M. (2021). Performance Analysis of Conventional Machine Learning Algorithms for Diabetic Sensorimotor Polyneuropathy Severity Classification. Diagnostics.

[3] Roglic G. (2016). WHO Global report on diabetes: A summary. Int. J. Noncommun. Dis..

[4] Costacou T., Orchard T.J. (2017). Cumulative Kidney Complication Risk by 50 Years of Type 1 Diabetes: The Effects of Sex, Age, and Calendar Year at Onset. Diabetes Care.

[5] Saran R., Robinson B., Abbott K.C., Agodoa L.Y., Albertus P., Ayanian J., Balkrishnan R., Bragg-Gresham J., Cao J., Chen J.L.T. (2017). US Renal Data System 2016 Annual Data Report: Epidemiology of Kidney Disease in the United States. Am. J. Kidney Dis..

[6] Colombo M., McGurnaghan S.J., Bell S., MacKenzie F., Patrick A.W., Petrie J.R., McKnight J.A., MacRury S., Traynor J., Metcalfe W. (2020). Predicting renal disease progression in a large contemporary cohort with type 1 diabetes mellitus. Diabetologia.

[7] LeCaire T.J., Klein B.E., Howard K.P., Lee K.E., Klein R. (2013). Risk for End-Stage Renal Disease Over 25 Years in the Population-Based WESDR Cohort. Diabetes Care.

[8] Vistisen D., Andersen G.S., Hulman A., McGurnaghan S., Colhoun H.M., Henriksen J.E., Thomsen R.W., Persson F., Rossing P., Jørgensen M.E. (2020). 1615-P: Predicting End-Stage Kidney Disease in Type 1 Diabetes. Diabetes.

[9] Helve J., Sund R., Arffman M., Harjutsalo V., Groop P.-H., Grönhagen-Riska C., Finne P. (2017). Incidence of End-Stage Renal Disease in Patients With Type 1 Diabetes. Diabetes Care.

[10] Webster A.C., Nagler E.V., Morton R.L., Masson P. (2017). Chronic Kidney Disease. Lancet.

[11] Levey A.S., Becker C., Inker L.A. (2015). Glomerular Filtration Rate and Albuminuria for Detection and Staging of Acute and Chronic Kidney Disease in Adults. JAMA.

[12] The Kidney Disease: Improving Global Outcomes (KDIGO) CKD Evaluation and 461 Management. https://kdigo.org/guidelines/ckd-evaluation-and-management/.

[13] Segal Z., Kalifa D., Radinsky K., Ehrenberg B., Elad G., Maor G., Lewis M., Tibi M., Korn L., Koren G. (2020). Machine learning algorithm for early detection of end-stage renal disease. BMC Nephrol..

[14] Cassia M.A., Pozzi F.E., Bascapè S., Saggiante L., Daminelli G., Cirelli C., Andi P.T.D., Elli M., Gallieni M. (2016). Proteinuria and Albuminuria at Point of Care. Nephrol. Point Care.

[15] National Institute for Health and Care Excellence (NICE) (2021). Chronic Kidney Disease: Assessment and Management NICE Guideline. https://www.nice.org.uk/guidance/ng203.

[16] Haque F., Reaz M.B.I., Chowdhury M.E.H., Hashim F.H., Arsad N., Ali S.H.M. (2021). Diabetic Sensorimotor Polyneuropathy Severity Classification Using Adaptive Neuro Fuzzy Inference System. IEEE Access.

[17] Islam J., Ahmad S., Haque F., Reaz M.B.I., Bhuiyan M.A.S., Islam R. (2021). A Novel Signal Normalization Approach to Improve the Force Invariant Myoelectric Pattern Recognition of Transradial Amputees. IEEE Access.

[18] Islam J., Ahmad S., Haque F., Reaz M., Bhuiyan M. (2021). Islam Force-Invariant Improved Feature Extraction Method for Upper-Limb Prostheses of Transradial Amputees. Diagnostics.

[19] Dagliati A., Marini S., Sacchi L., Cogni G., Teliti M., Tibollo V., De Cata P., Chiovato L., Bellazzi R. (2017). Machine Learning Methods to Predict Diabetes Complications. J. Diabetes Sci. Technol..

[20] Xiao J., Ding R., Xu X., Guan H., Feng X., Sun T., Zhu S., Ye Z. (2019). Comparison and development of machine learning tools in the prediction of chronic kidney disease progression. J. Transl. Med..

[21] Jeong B., Cho H., Kim J., Kil Kwon S., Hong S., Lee C., Kim T., Park M.S., Hong S., Heo T.-Y. (2020). Comparison between Statistical Models and Machine Learning Methods on Classification for Highly Imbalanced Multiclass Kidney Data. Diagnostics.

[22] Makino M., Ono M., Itoko T., Katsuki T., Koseki A., Kudo M., Haida K., Kuroda J., Yanagiya R., Suzuki A. (2018). Artificial Intelligence Predicts Progress of Diabetic Kidney Disease-Novel Prediction Model Construction with Big Data Machine Learning. Diabetes.

[23] Low S., Lim S.C., Zhang X., Zhou S., Yeoh L.Y., Liu Y.L., Tavintharan S., Sum C.F. (2017). Development and validation of a predictive model for Chronic Kidney Disease progression in Type 2 Diabetes Mellitus based on a 13-year study in Singapore. Diabetes Res. Clin. Pract..

[24] Chien K.-L., Lin H.-J., Lee B.-C., Hsu H.-C., Lee Y.-T., Chen M.-F. (2010). A Prediction Model for the Risk of Incident Chronic Kidney Disease. Am. J. Med..

[25] Dunkler D., Gao P., Lee S.F., Heinze G., Clase C.M., Tobe S., Teo K., Gerstein H., Mann J.F., Oberbauer R. (2015). Risk Prediction for Early CKD in Type 2 Diabetes. Clin. J. Am. Soc. Nephrol..

[26] Aspriello S.D., Zizzi A., Tirabassi G., Buldreghini E., Biscotti T., Faloia E., Stramazzotti D., Boscaro M., Piemontese M. (2010). Diabetes mellitus-associated periodontitis: Differences between type 1 and type 2 diabetes mellitus. J. Periodontal Res..

[27] Lee Y.-B., Han K., Kim B., Jun J.E., Lee S.-E., Ahn J., Kim G., Jin S.-M., Kim J.H. (2019). Risk of end-stage renal disease from chronic kidney disease defined by decreased glomerular filtration rate in type 1 diabetes: A comparison with type 2 diabetes and the effect of metabolic syndrome. Diabetes Metab. Res. Rev..

[28] Kristófi R., Bodegard J., Norhammar A., Thuresson M., Nathanson D., Nyström T., Birkeland K.I., Eriksson J.W. (2021). Cardiovascular and Renal Disease Burden in Type 1 Compared With Type 2 Diabetes: A Two-Country Nationwide Observational Study. Diabetes Care.

[29] National Kidney Foundation (2021). “ACR”. https://www.kidney.org/kidneydisease/siemens_hcp_acr.

[30] Epidemiology of Diabetes Interventions and Complications (EDIC) (1999). Design, implementation, and preliminary results of a long-term follow-up of the Diabetes Control and Complications Trial cohort. Diabetes Care.

[31] The DCCT/EDIC Research Group (2011). Intensive Diabetes Therapy and Glomerular Filtration Rate in Type 1 Diabetes. N. Engl. J. Med..

[32] National Institute of Diabetes and Digestive and Kidney Diseases (NIDDK) (2021). Epidemiology of Diabetes Interventions and Complications (EDIC). https://www.clinicaltrials.gov/ct2/show/NCT00360893.

[33] Molitch M.E., Steffes M., Sun W., Rutledge B., Cleary P., de Boer I.H., Zinman B., Lachin J. (2010). Development and Progression of Renal Insufficiency With and Without Albuminuria in Adults With Type 1 Diabetes in the Diabetes Control and Complications Trial and the Epidemiology of Diabetes Interventions and Complications Study. Diabetes Care.

[34] Fabiny D.L., Ertingshausen G. (1971). Automated Reaction-Rate Method for Determination of Serum Creatinine with the CentrifiChem. Clin. Chem..

[35] Silveiro S.P., Araújo G.N., Ferreira M.N., Souza F.D., Yamaguchi H.M., Camargo E.G. (2011). Chronic Kidney Disease Epidemiology Collaboration (CKD-EPI) Equation Pronouncedly Underestimates Glomerular Filtration Rate in Type 2 Diabetes: Figure. Diabetes Care.

[36] Perkins B.A., Bebu I., de Boer I.H., Molitch M., Tamborlane W., Lorenzi G., Herman W., White N.H., Pop-Busui R., Paterson A.D. (2019). Risk Factors for Kidney Disease in Type 1 Diabetes. Diabetes Care.

[37] De Boer I.H., Rue T.C., Cleary P.A., Lachin J., Molitch M.E., Steffes M.W., Sun W., Zinman B., Brunzell J.D. (2011). Diabetes Control and Complications Trial/Epidemiology of Diabetes Interventions and Complications Study Research Group. Long-Term Renal Outcomes of Patients with Type 1 Diabetes Mellitus and Microalbuminuria: An Analysis of the Diabetes Control and Complications Trial/Epidemiology of Diabetes Interventions and Complications Cohort Microalbuminuria Outcomes in Type 1 Diabetes. Arch. Intern. Med..

[38] Radcliffe N.J., Seah J.M., Clarke M., MacIsaac R.J., Jerums G., Ekinci E.I. (2016). Clinical predictive factors in diabetic kidney disease progression. J. Diabetes Investig..

[39] Tangri N., Kitsios G.D., Inker L.A., Griffith J., Naimark D.M., Walker S., Rigatto C., Uhlig K., Kent D.M., Levey A.S. (2013). Risk Prediction Models for Patients With Chronic Kidney Disease. Ann. Intern. Med..

[40] Papadopoulou-Marketou N., Chrousos G.P., Kanaka-Gantenbein C. (2016). Diabetic nephropathy in type 1 diabetes: A review of early natural history, pathogenesis, and diagnosis. Diabetes Metab. Res. Rev..

[41] Liu Y., Wang Y., Zhang J. (2012). New Machine Learning Algorithm: Random Forest. Information Computing and Applications.

[42] Peterson L.E. (2009). K-nearest neighbor. Scholarpedia.

[43] Azur M.J., Stuart E., Frangakis C., Leaf P.J. (2011). Multiple imputation by chained equations: What is it and how does it work?. Int. J. Methods Psychiatr. Res..

[44] Chawla N.V., Bowyer K.W., Hall L.O., Kegelmeyer W.P. (2002). SMOTE: Synthetic Minority Over-sampling Technique. J. Artif. Intell. Res..

[45] Goel G., Maguire L., Li Y., McLoone S. (2013). Evaluation of Sampling Methods for Learning from Imbalanced Data. Proceedings of the Intelligent Computing Theories.

[46] Chen T., Guestrin C. XGBoost: A Scalable Tree Boosting System. Proceedings of the 22nd ACM SIGKDD International Conference on Knowledge Discovery and Data Mining.

[47] Geurts P., Ernst D., Wehenkel L. (2006). Extremely randomized trees. Mach. Learn..

[48] Scikit-Learn: Machine Learning in Python—Scikit-Learn 0.24.2 Documentation. https://scikit-learn.org/stable/index.html.

[49] Haque F., Reaz M.B.I., Chowdhury M., Ali S.H.M., Bakar A., Rahman T., Kobashi S., Dhawale C., Bhuiyan M. (2021). A nomogram-based diabetic sensorimotor polyneuropathy severity prediction using Michigan neuropathy screening instrumentations. Comput. Biol. Med..

[50] Li H., Yu C., Chen R., Li J., Li J. (2012). Novel ionic liquid-type Gemini surfactants: Synthesis, surface property and antimicrobial activity. Colloids Surf. A Physicochem. Eng. Asp..

[51] Wang Z., Wu C., Zhe W., Niu X., Wang X. (2019). SMOTETomek-Based Resampling for Personality Recognition. IEEE Access.

