# Supplementary material for: Performance Analysis of Conventional Machine Learning Algorithms for Identification of Chronic Kidney Disease in Type 1 Diabetes Mellitus Patients
Source: Diagnostics (Basel). 2021 Dec 3;11(12):2267. doi: 10.3390/diagnostics11122267 (PMC8700037; doi:10.3390/diagnostics11122267)
Supplement: Supplementary file 1 [file diagnostics-11-02267-s001.zip › diagnostics-1440750-supplementary.pdf]

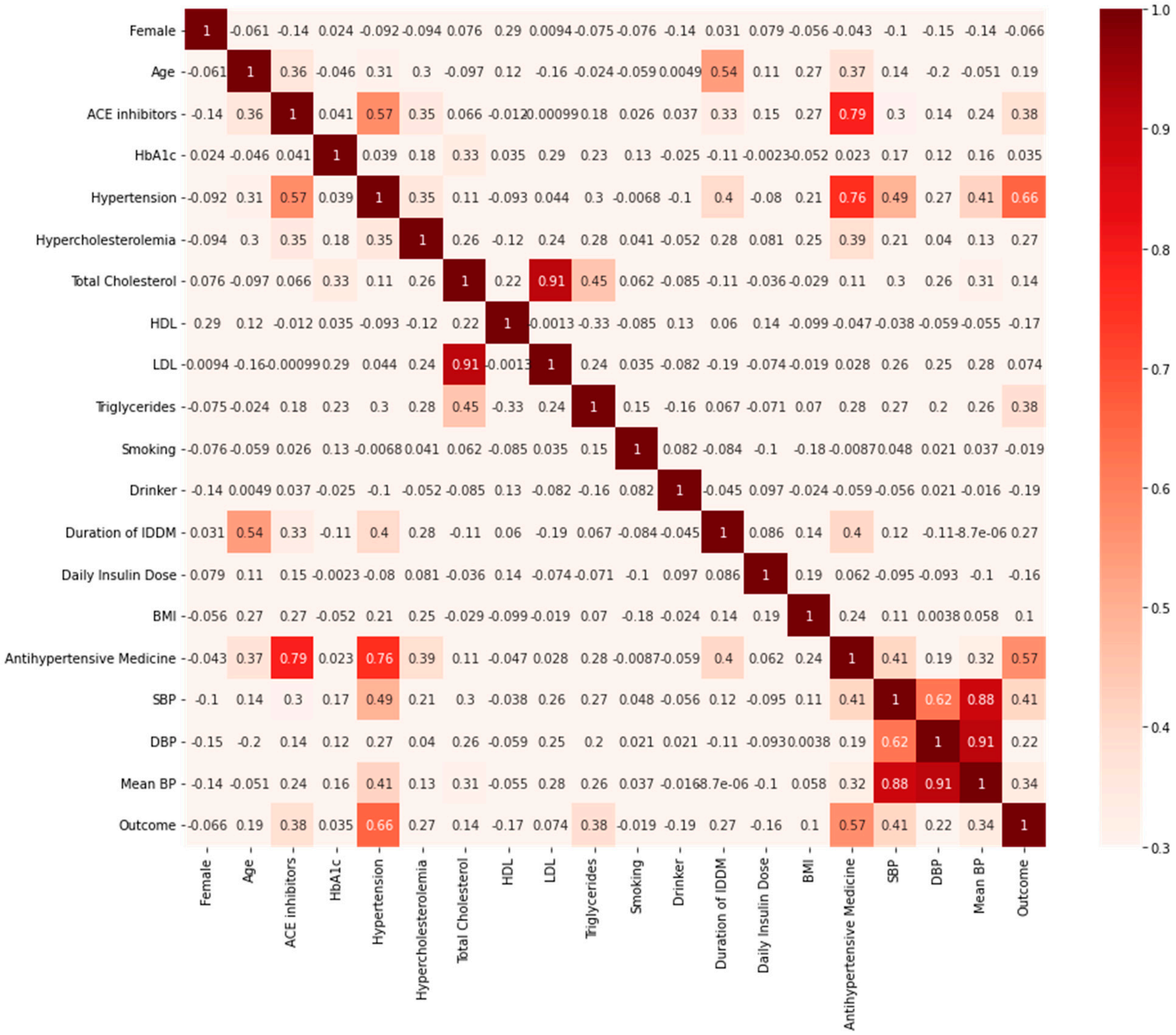

Figure S1: Features correlation heatmap of Dataset RF.

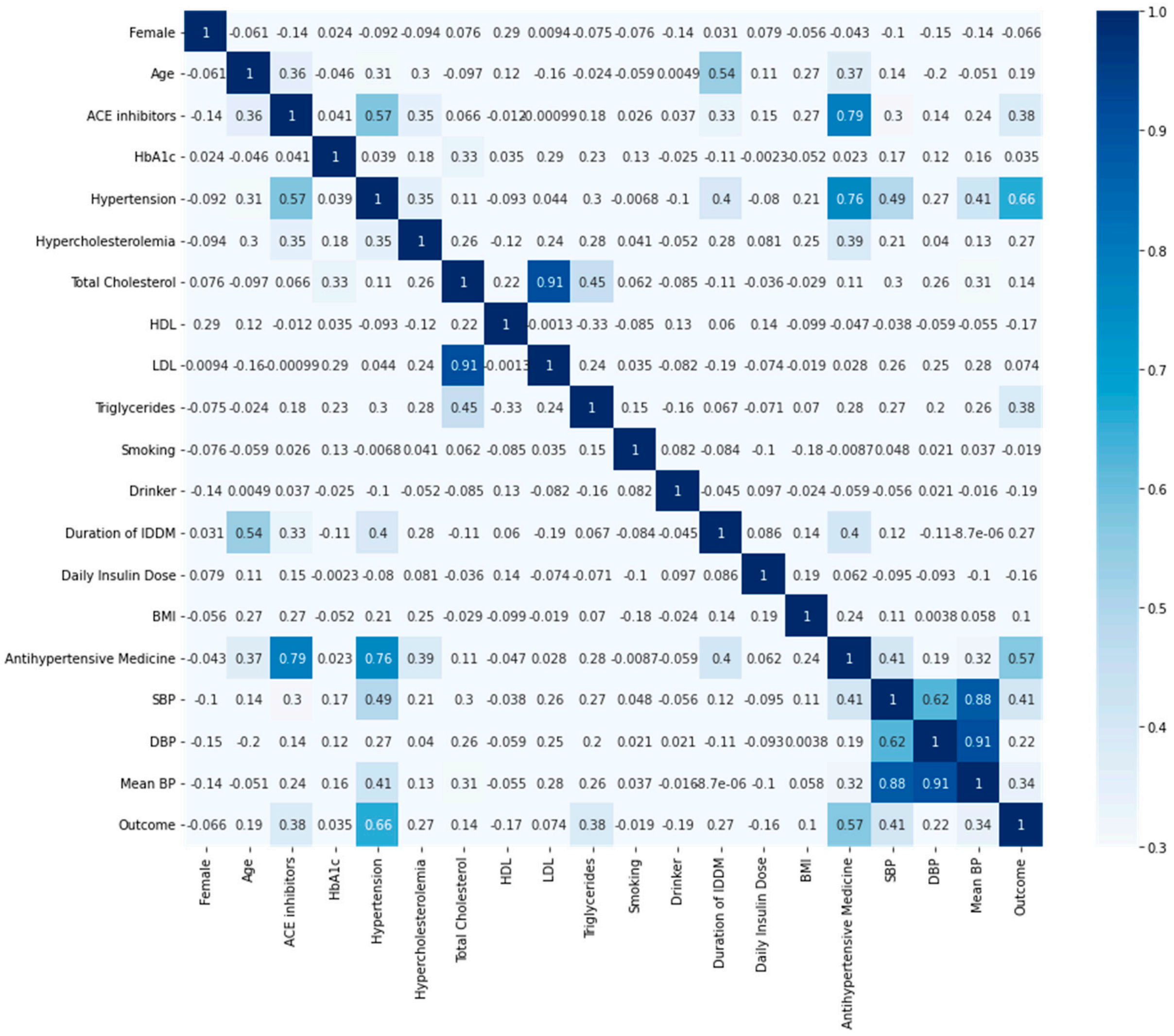

Figure S2: Features correlation heatmap of Dataset MICE.

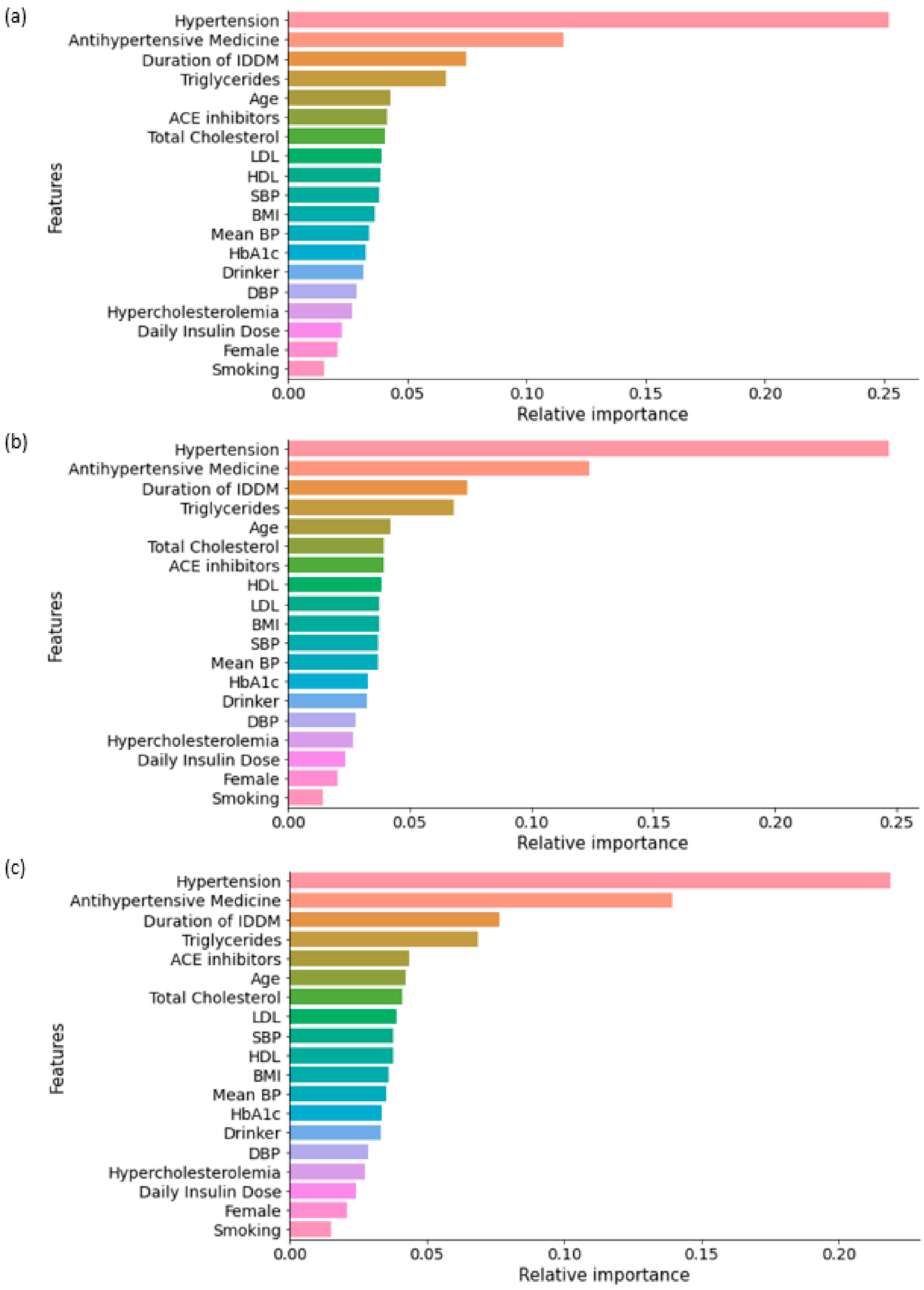

Figure S3: Features ranking using Extra tree algorithm. (a) on Dataset KNN (b) on Dataset MICE (c) on Dataset RF.

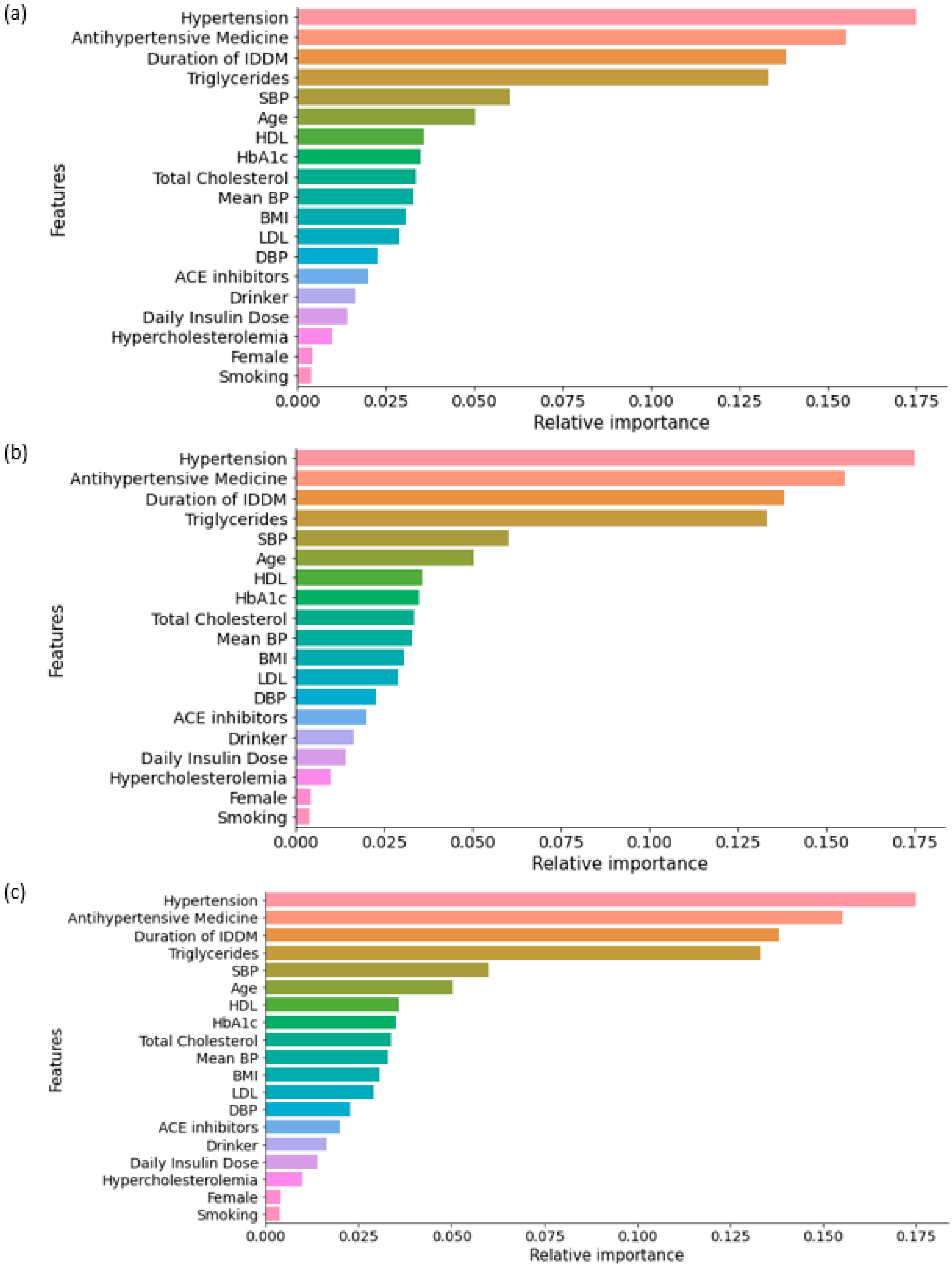

Figure S4: Features ranking using RF algorithm. (a) on Dataset KNN (b) on Dataset MICE (c) on Dataset RF.

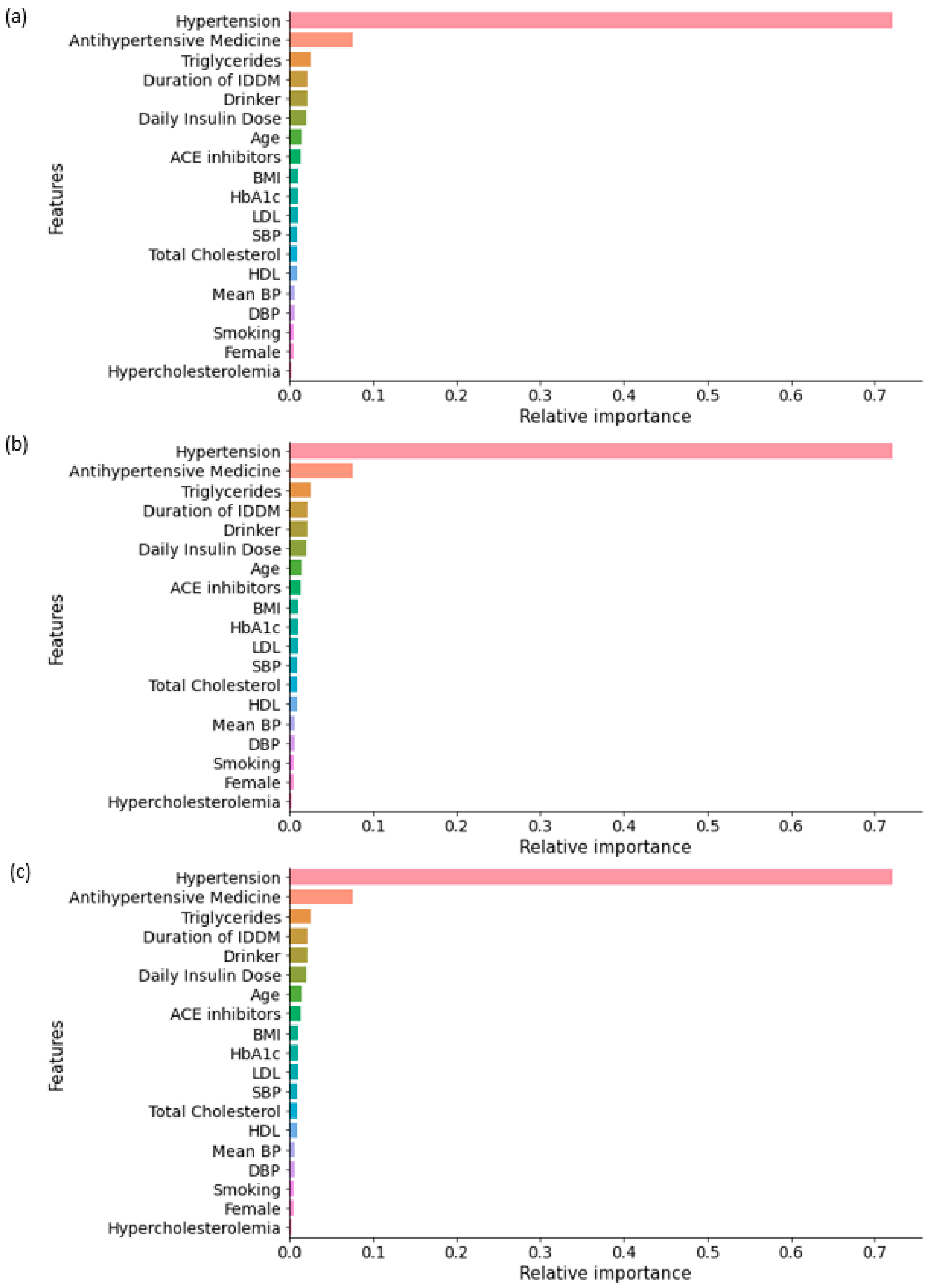

Figure S5: Features ranking using XGB algorithm. (a) on Dataset KNN (b) on Dataset MICE (c) on Dataset RF.

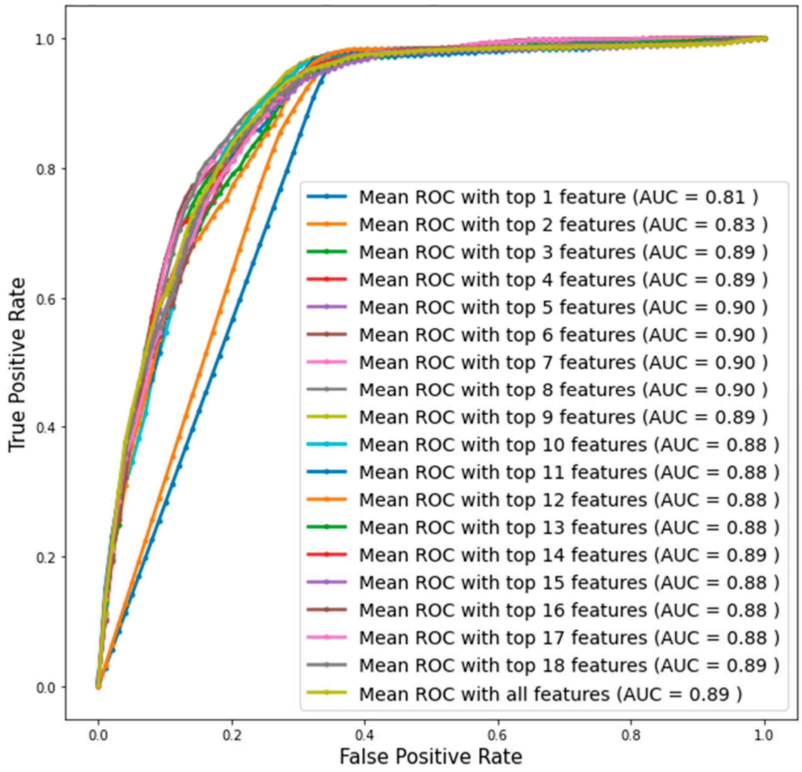

Figure S6: ROC curve of LR model with different features ranked by Extra Tree on Dataset KNN.

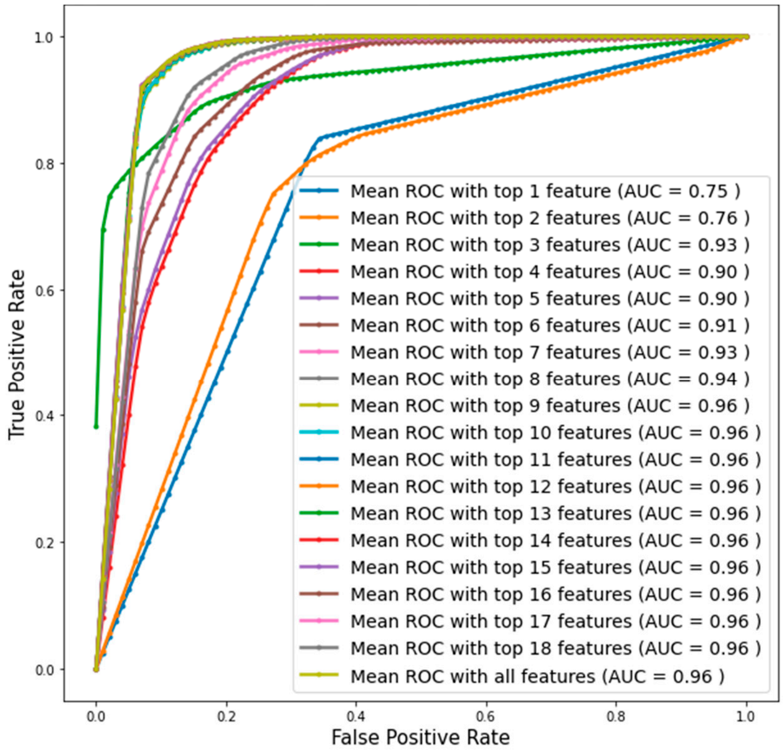

Figure S7: ROC curve of KNN model with different features ranked by Extra Tree on Dataset KNN.

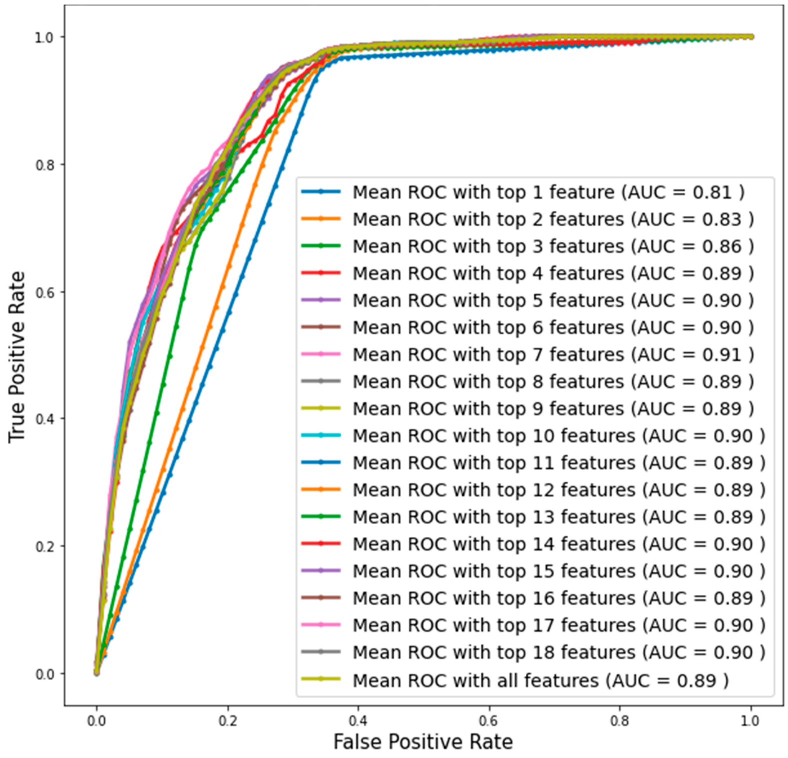

Figure S8: ROC curve of GNB model with different features ranked by XGB on Dataset KNN.

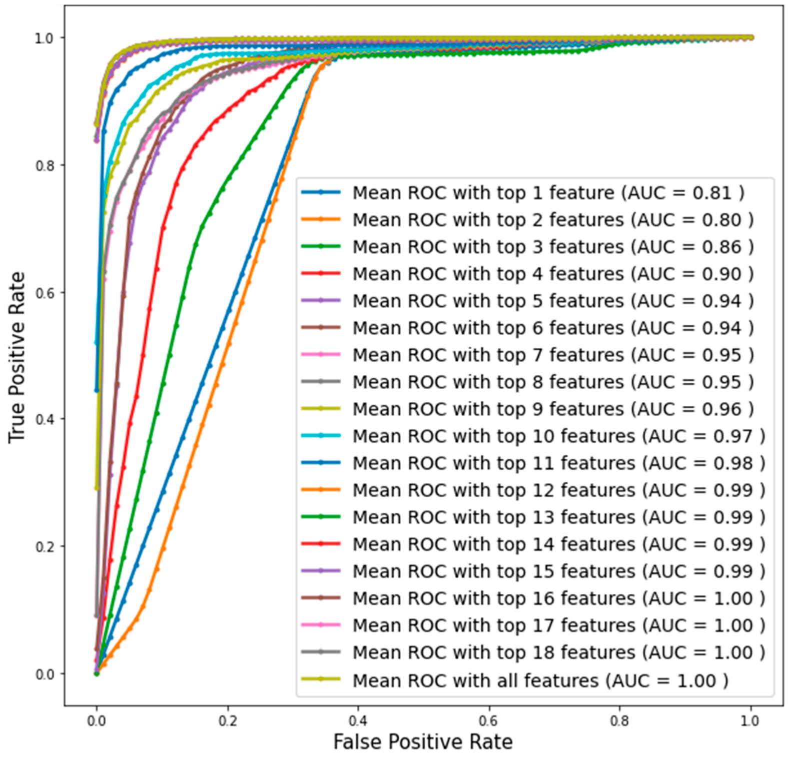

Figure S9: ROC curve of SVM model with different features ranked by XGB on Dataset KNN.

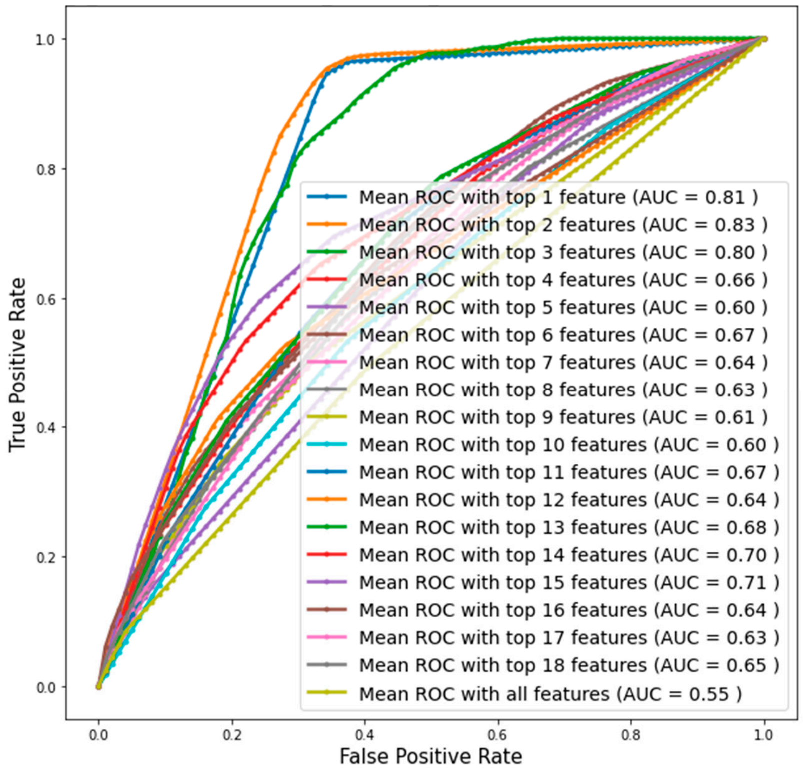

Figure S10: ROC curve of SGD model with different features ranked by Extra Tree on Dataset KNN.

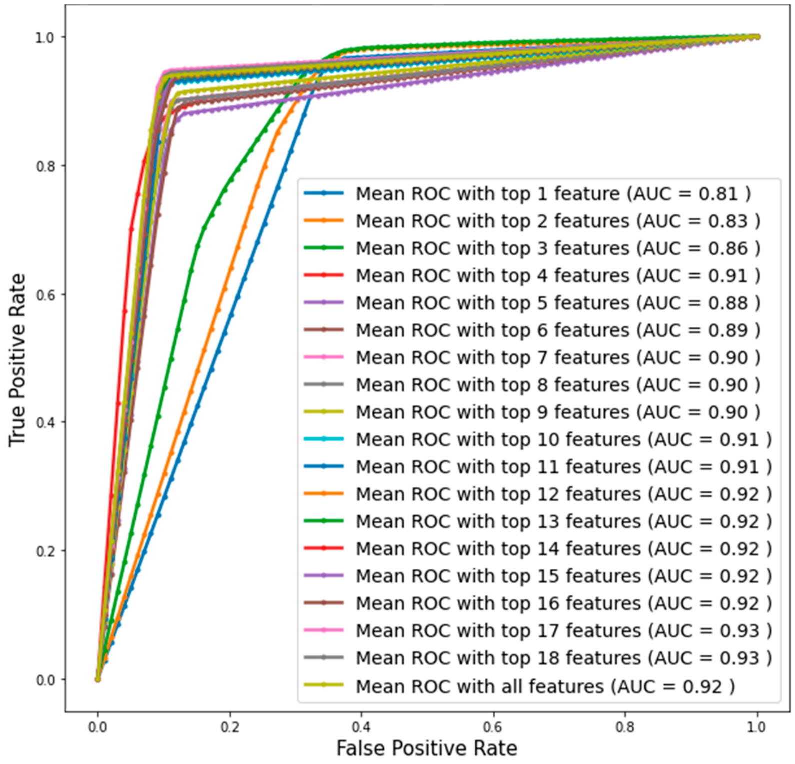

Figure S11: ROC curve of DT model with different features ranked by XGB on Dataset KNN.

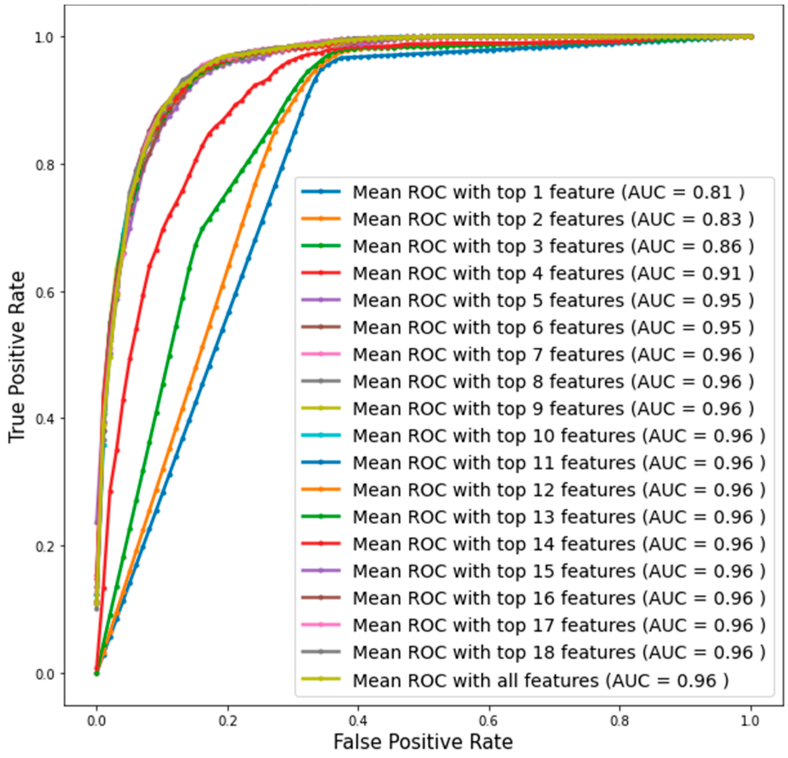

Figure S12: ROC curve of GB model with different features ranked by XGB on Dataset KNN.

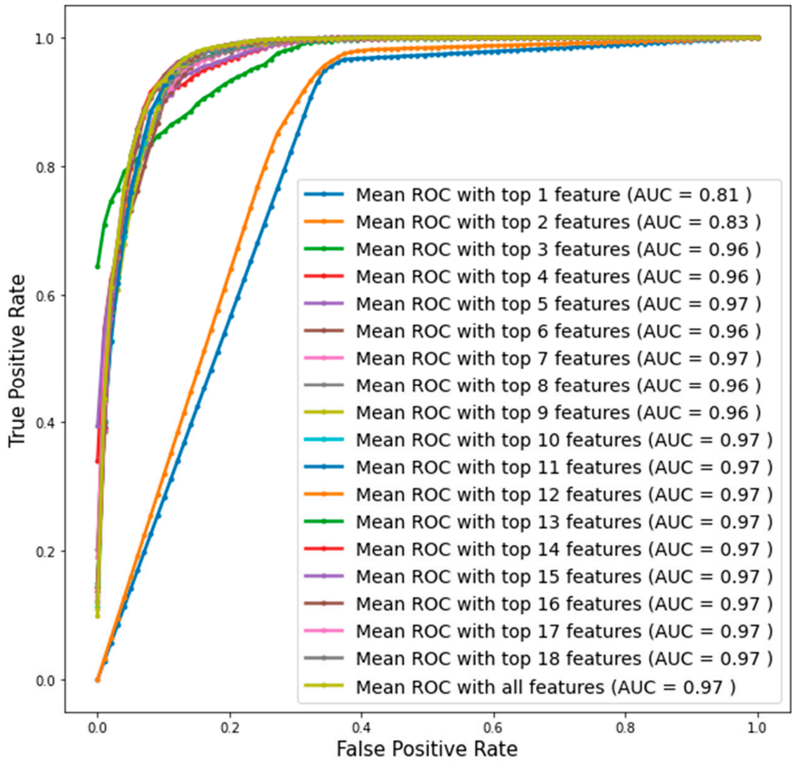

Figure S13: ROC curve of XGB model with different features ranked by Extra Tree on Dataset KNN.

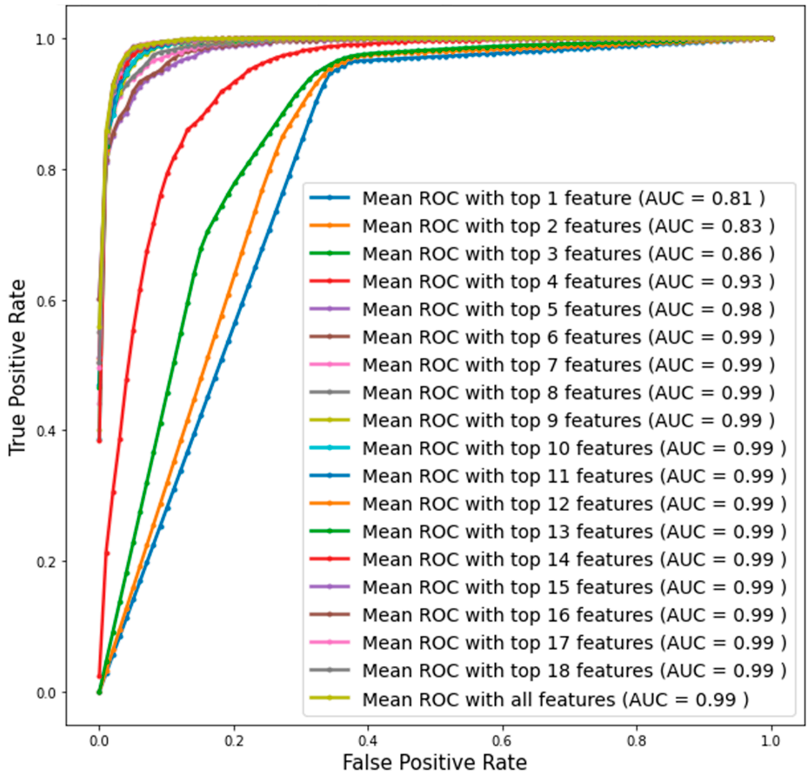

Figure S14: ROC curve of LightGMB model with different features ranked by XGB on Dataset KNN.

Table S1. Performance analysis of LR algorithm using KNN data imputation and Extra Tree feature ranking.

Number of Features	Sensitivity (Recall)	Specificity	Accuracy	Precision	F1_Score	Non-CKD		CKD	
						True Negative	False Positive	False Negative	True Positive
1	0.97(±0.01)	0.66(±0.03)	0.81(±0.01)	0.74(±0.02)	0.84(±0.01)	1822	959	91	2699
2	0.97(±0.01)	0.66(±0.03)	0.81(±0.01)	0.74(±0.02)	0.84(±0.01)	1822	959	91	2699
3	0.94(±0.01)	0.70(±0.04)	0.82(±0.01)	0.76(±0.02)	0.84(±0.01)	1939	842	171	2619
4	0.94(±0.01)	0.70(±0.04)	0.82(±0.01)	0.76(±0.02)	0.84(±0.01)	1946	835	170	2620
5	0.94(±0.01)	0.70(±0.04)	0.82(±0.01)	0.76(±0.02)	0.84(±0.01)	1953	828	170	2620
6	0.94(±0.01)	0.70(±0.04)	0.82(±0.01)	0.76(±0.02)	0.84(±0.01)	1949	832	170	2620
7	0.92(±0.03)	0.72(±0.04)	0.82(±0.02)	0.77(±0.02)	0.83(±0.02)	1997	784	229	2561
8	0.93(±0.04)	0.72(±0.04)	0.83(±0.01)	0.77(±0.02)	0.84(±0.01)	1999	782	187	2603
9	0.93(±0.05)	0.73(±0.05)	0.83(±0.01)	0.78(±0.03)	0.84(±0.01)	2029	752	200	2590
10	0.93(±0.04)	0.73(±0.05)	0.83(±0.01)	0.77(±0.03)	0.84(±0.01)	2027	754	204	2586
11	0.89(±0.08)	0.73(±0.05)	0.81(±0.02)	0.77(±0.02)	0.82(±0.03)	2036	745	316	2474
12	0.86(±0.05)	0.74(±0.03)	0.80(±0.02)	0.77(±0.02)	0.81(±0.02)	2065	716	385	2405
13	0.87(±0.04)	0.75(±0.05)	0.81(±0.02)	0.78(±0.03)	0.82(±0.02)	2076	705	360	2430
14	0.87(±0.03)	0.76(±0.04)	0.81(±0.02)	0.78(±0.03)	0.82(±0.01)	2110	671	362	2428
15	0.87(±0.03)	0.76(±0.04)	0.81(±0.01)	0.78(±0.02)	0.82(±0.01)	2114	667	373	2417
16	0.87(±0.03)	0.76(±0.04)	0.82(±0.02)	0.79(±0.03)	0.83(±0.02)	2121	660	361	2429
17	0.87(±0.02)	0.76(±0.03)	0.81(±0.02)	0.79(±0.02)	0.82(±0.02)	2121	660	376	2414
18	0.87(±0.04)	0.76(±0.04)	0.82(±0.02)	0.79(±0.03)	0.83(±0.02)	2122	659	364	2426
19	0.88(±0.06)	0.77(±0.06)	0.82(±0.02)	0.79(±0.03)	0.83(±0.02)	2131	650	339	2451

Table S2. Performance analysis of KNN algorithm using KNN data imputation and Extra Tree feature ranking.

Number of Features	Sensitivity (Recall)	Specificity	Accuracy	Precision	F1_Score	Non-CKD		CKD	
						True Negative	False Positive	False Negative	True Positive
1	0.58(±0.94)	0.80(±0.33)	0.69(±0.31)	0.44(±0.73)	0.50(±0.82)	2220	562	118<br>0	1610
2	0.52(±0.85)	0.84(±0.26)	0.68(±0.29)	0.46(±0.75)	0.49(±0.80)	2342	440	134<br>6	1444
3	0.80(±0.46)	0.92(±0.08)	0.86(±0.20)	0.91(±0.06)	0.83(±0.33)	2562	220	566	2224
4	0.91(±0.03)	0.74(±0.04)	0.83(±0.02)	0.78(±0.03)	0.84(±0.02)	2072	710	251	2539
5	0.91(±0.03)	0.75(±0.03)	0.83(±0.03)	0.79(±0.02)	0.84(±0.02)	2094	688	248	2542
6	0.93(±0.02)	0.76(±0.02)	0.84(±0.02)	0.79(±0.02)	0.86(±0.02)	2111	671	194	2596
7	0.95(±0.01)	0.79(±0.01)	0.87(±0.01)	0.82(±0.01)	0.88(±0.01)	2184	598	128	2662
8	0.97(±0.01)	0.77(±0.02)	0.87(±0.01)	0.81(±0.01)	0.88(±0.01)	2152	630	78	2712
9	0.99(±0.02)	0.81(±0.03)	0.90(±0.01)	0.84(±0.02)	0.91(±0.01)	2240	542	33	2757
10	0.99(±0.01)	0.80(±0.04)	0.90(±0.02)	0.83(±0.03)	0.90(±0.01)	2223	559	26	2764
11	0.99(±0.01)	0.80(±0.04)	0.90(±0.02)	0.83(±0.03)	0.91(±0.01)	2230	552	24	2766
12	0.99(±0.01)	0.80(±0.04)	0.90(±0.02)	0.83(±0.03)	0.91(±0.01)	2229	553	24	2766
13	0.99(±0.01)	0.80(±0.04)	0.90(±0.02)	0.83(±0.03)	0.91(±0.01)	2230	552	24	2766
14	0.99(±0.01)	0.80(±0.04)	0.90(±0.02)	0.83(±0.03)	0.91(±0.02)	2230	552	19	2771
15	0.99(±0.01)	0.80(±0.04)	0.90(±0.02)	0.83(±0.03)	0.91(±0.02)	2230	552	19	2771
16	0.99(±0.01)	0.80(±0.04)	0.90(±0.02)	0.83(±0.03)	0.90(±0.01)	2216	566	19	2771
17	0.99(±0.01)	0.80(±0.04)	0.90(±0.02)	0.83(±0.03)	0.90(±0.01)	2216	566	19	2771
18	0.99(±0.01)	0.80(±0.04)	0.90(±0.02)	0.83(±0.03)	0.90(±0.01)	2217	565	19	2771
19	0.99(±0.01)	0.80(±0.04)	0.90(±0.02)	0.83(±0.03)	0.90(±0.01)	2217	565	19	2771

Table S3. Performance analysis of GNB algorithm using KNN data imputation and XGB feature ranking.

Number of Features	Sensitivity (Recall)	Specificity	Accuracy	Precision	F1_Score	Non-CKD		CKD	
						True Negative	False Positive	False Negative	True Positive
1	0.96(±0.01)	0.66(±0.03)	0.81(±0.01)	0.74(±0.02)	0.84(±0.01)	1824	958	100	2690
2	0.87(±0.02)	0.73(±0.04)	0.80(±0.02)	0.76(±0.03)	0.81(±0.02)	2017	765	374	2416
3	0.87(±0.02)	0.73(±0.04)	0.80(±0.02)	0.76(±0.03)	0.81(±0.02)	2017	765	374	2416
4	0.88(±0.02)	0.72(±0.04)	0.80(±0.02)	0.76(±0.03)	0.82(±0.02)	2006	776	333	2457
5	0.88(±0.03)	0.74(±0.04)	0.81(±0.02)	0.77(±0.03)	0.82(±0.01)	2059	723	325	2465
6	0.89(±0.03)	0.74(±0.04)	0.82(±0.02)	0.77(±0.03)	0.83(±0.02)	2057	725	298	2492
7	0.93(±0.02)	0.74(±0.04)	0.83(±0.02)	0.78(±0.03)	0.85(±0.01)	2056	726	198	2592
8	0.92(±0.03)	0.74(±0.04)	0.83(±0.02)	0.78(±0.02)	0.84(±0.02)	2047	735	234	2556
9	0.92(±0.03)	0.74(±0.04)	0.83(±0.02)	0.78(±0.02)	0.84(±0.01)	2049	733	224	2566
10	0.92(±0.03)	0.74(±0.04)	0.83(±0.02)	0.78(±0.03)	0.84(±0.02)	2047	735	223	2567
11	0.92(±0.03)	0.74(±0.04)	0.83(±0.02)	0.78(±0.03)	0.84(±0.02)	2048	734	235	2555
12	0.92(±0.03)	0.74(±0.04)	0.83(±0.02)	0.78(±0.03)	0.84(±0.02)	2047	735	233	2557
13	0.92(±0.03)	0.74(±0.04)	0.83(±0.02)	0.78(±0.03)	0.85(±0.02)	2051	731	214	2576
14	0.93(±0.02)	0.74(±0.05)	0.83(±0.02)	0.78(±0.03)	0.85(±0.01)	2066	716	206	2584
15	0.93(±0.03)	0.75(±0.05)	0.84(±0.02)	0.79(±0.03)	0.85(±0.01)	2074	708	203	2587
16	0.91(±0.02)	0.75(±0.05)	0.83(±0.02)	0.78(±0.03)	0.84(±0.01)	2083	699	255	2535
17	0.90(±0.02)	0.75(±0.04)	0.83(±0.02)	0.78(±0.03)	0.84(±0.01)	2082	700	268	2522
18	0.90(±0.02)	0.75(±0.05)	0.83(±0.02)	0.78(±0.03)	0.84(±0.01)	2084	698	274	2516
19	0.90(±0.02)	0.75(±0.04)	0.82(±0.02)	0.78(±0.02)	0.84(±0.01)	2087	695	286	2504

Table S4. Performance analysis of SVM algorithm using KNN data imputation and XGB feature ranking.

Number of Features	Sensitivity (Recall)	Specificity	Accuracy	Precision	F1_Score	Non-CKD		CKD	
						True Negative	False Positive	False Negative	True Positive
1	0.97(±0.00)	0.66(±0.03)	0.81(±0.02)	0.74(±0.02)	0.84(±0.01)	1825	957	86	2704
2	0.97(±0.00)	0.66(±0.03)	0.81(±0.02)	0.74(±0.02)	0.84(±0.01)	1825	957	86	2704
3	0.96(±0.01)	0.68(±0.04)	0.82(±0.02)	0.75(±0.02)	0.84(±0.01)	1895	887	115	2675
4	0.90(±0.02)	0.78(±0.03)	0.84(±0.02)	0.80(±0.02)	0.85(±0.01)	2170	612	269	2521
5	0.93(±0.03)	0.83(±0.04)	0.88(±0.02)	0.85(±0.03)	0.89(±0.01)	2315	467	205	2585
6	0.94(±0.02)	0.83(±0.04)	0.88(±0.01)	0.85(±0.03)	0.89(±0.01)	2316	466	179	2611
7	0.88(±0.04)	0.89(±0.02)	0.89(±0.01)	0.89(±0.02)	0.89(±0.01)	2481	301	329	2461
8	0.89(±0.04)	0.89(±0.02)	0.89(±0.01)	0.89(±0.02)	0.89(±0.01)	2463	319	307	2483
9	0.88(±0.06)	0.94(±0.01)	0.91(±0.02)	0.93(±0.01)	0.90(±0.03)	2603	179	335	2455
10	0.78(±0.07)	0.99(±0.01)	0.88(±0.03)	0.98(±0.01)	0.87(±0.04)	2741	41	627	2163
11	0.71(±0.09)	1.00(±0.00)	0.85(±0.05)	1.00(±0.00)	0.83(±0.06)	2780	2	820	1970
12	0.69(±0.09)	1.00(±0.00)	0.84(±0.05)	1.00(±0.00)	0.81(±0.07)	2782	0	874	1916
13	0.69(±0.09)	1.00(±0.00)	0.85(±0.05)	1.00(±0.00)	0.82(±0.06)	2782	0	853	1937
14	0.68(±0.10)	1.00(±0.00)	0.84(±0.05)	1.00(±0.00)	0.81(±0.07)	2782	0	890	1900
15	0.69(±0.10)	1.00(±0.00)	0.84(±0.05)	1.00(±0.00)	0.82(±0.07)	2782	0	866	1924
16	0.67(±0.11)	1.00(±0.00)	0.84(±0.06)	1.00(±0.00)	0.80(±0.08)	2782	0	915	1875
17	0.68(±0.10)	1.00(±0.00)	0.84(±0.05)	1.00(±0.00)	0.81(±0.07)	2782	0	886	1904
18	0.69(±0.10)	1.00(±0.00)	0.84(±0.05)	1.00(±0.00)	0.81(±0.07)	2782	0	869	1921
19	0.69(±0.10)	1.00(±0.00)	0.84(±0.05)	1.00(±0.00)	0.81(±0.07)	2782	0	872	1918

Table S5. Performance analysis of SGD algorithm using KNN data imputation and Extra Tree feature ranking.

Number of Features	Sensitivity (Recall)	Specificity	Accuracy	Precision	F1_Score	Non-CKD		CKD	
						True Negative	False Positive	False Negative	True Positive
1	0.37(±0.90)	0.82(±0.30)	0.69(±0.31)	0.60(±0.60)	0.66(±0.66)	1744	1039	137	2653
2	0.96(±0.01)	0.74(±0.27)	0.80(±0.02)	0.59(±0.59)	0.49(±0.80)	1860	923	159	2631
3	0.96(±0.01)	0.65(±0.03)	0.81(±0.02)	0.74(±0.02)	0.83(±0.01)	2013	770	642	2148
4	0.32(±0.74)	0.43(±0.75)	0.69(±0.22)	0.62(±0.68)	0.32(±0.68)	1252	1531	331	2459
5	0.79(±0.79)	0.88(±0.19)	0.63(±0.17)	0.44(±0.47)	0.61(±0.47)	1881	902	931	1859
6	0.87(±0.51)	0.51(±0.66)	0.53(±0.07)	0.57(±0.61)	0.51(±0.56)	1535	1248	1164	1626
7	0.74(±0.65)	0.66(±0.63)	0.62(±0.07)	0.71(±0.22)	0.60(±0.39)	2272	511	1819	971
8	0.79(±0.79)	0.65(±0.69)	0.66(±0.14)	0.70(±0.20)	0.53(±0.28)	1612	1171	1312	1478
9	0.91(±0.19)	0.60(±0.79)	0.59(±0.11)	0.72(±0.24)	0.62(±0.25)	903	1880	329	2461
10	0.61(±0.95)	0.64(±0.62)	0.59(±0.14)	0.71(±0.17)	0.45(±0.52)	1299	1484	446	2344
11	0.50(±0.55)	0.62(±0.82)	0.62(±0.14)	0.73(±0.18)	0.59(±0.28)	1429	1354	449	2341
12	0.91(±0.14)	0.92(±0.14)	0.65(±0.10)	0.73(±0.29)	0.36(±0.67)	1452	1331	708	2082
13	0.61(±0.73)	0.64(±0.71)	0.67(±0.07)	0.47(±0.49)	0.54(±0.38)	1602	1181	875	1915
14	0.88(±0.21)	0.74(±0.56)	0.61(±0.20)	0.70(±0.28)	0.56(±0.44)	1986	797	1051	1739
15	0.48(±0.62)	0.58(±0.65)	0.62(±0.19)	0.75(±0.27)	0.41(±0.56)	1975	808	1010	1780
16	0.73(±0.64)	0.89(±0.27)	0.65(±0.10)	0.48(±0.52)	0.53(±0.58)	1681	1102	1171	1619
17	0.60(±0.53)	0.64(±0.73)	0.62(±0.17)	0.76(±0.27)	0.58(±0.34)	2194	589	1384	1406
18	0.73(±0.65)	0.46(±0.48)	0.64(±0.23)	0.72(±0.19)	0.66(±0.21)	1749	1034	856	1934
19	0.49(±0.56)	0.62(±0.69)	0.63(±0.19)	0.64(±0.24)	0.67(±0.28)	2607	176	2079	711

Table S6. Performance analysis of DT algorithm using KNN data imputation and XGB feature ranking.

Number of Features	Sensitivity (Recall)	Specificity	Accuracy	Precision	F1_Score	Non-CKD		CKD	
						True Negative	False Positive	False Negative	True Positive
1	0.96(±0.01)	0.66(±0.03)	0.81(±0.01)	0.74(±0.02)	0.84(±0.01)	1824	958	100	2690
2	0.96(±0.01)	0.66(±0.03)	0.81(±0.01)	0.74(±0.02)	0.84(±0.01)	1824	958	100	2690
3	0.95(±0.01)	0.68(±0.04)	0.82(±0.01)	0.75(±0.02)	0.84(±0.01)	1894	888	132	2658
4	0.83(±0.13)	0.91(±0.03)	0.87(±0.06)	0.91(±0.02)	0.87(±0.08)	2539	243	462	2328
5	0.87(±0.16)	0.89(±0.02)	0.88(±0.08)	0.89(±0.02)	0.88(±0.10)	2487	295	355	2435
6	0.89(±0.16)	0.89(±0.03)	0.89(±0.07)	0.89(±0.01)	0.89(±0.08)	2472	310	305	2485
7	0.90(±0.15)	0.89(±0.02)	0.90(±0.06)	0.89(±0.02)	0.89(±0.08)	2482	300	286	2504
8	0.90(±0.14)	0.89(±0.02)	0.90(±0.07)	0.90(±0.02)	0.90(±0.08)	2499	283	271	2519
9	0.90(±0.13)	0.89(±0.01)	0.90(±0.06)	0.89(±0.01)	0.90(±0.07)	2492	290	262	2528
10	0.92(±0.08)	0.90(±0.01)	0.91(±0.04)	0.90(±0.01)	0.91(±0.04)	2507	275	203	2587
11	0.93(±0.06)	0.91(±0.02)	0.92(±0.03)	0.91(±0.01)	0.92(±0.03)	2513	269	179	2611
12	0.93(±0.05)	0.90(±0.02)	0.92(±0.03)	0.91(±0.02)	0.92(±0.03)	2514	268	183	2607
13	0.94(±0.04)	0.91(±0.01)	0.92(±0.02)	0.91(±0.01)	0.92(±0.02)	2507	275	178	2612
14	0.94(±0.04)	0.91(±0.01)	0.92(±0.02)	0.91(±0.02)	0.92(±0.02)	2517	265	177	2613
15	0.94(±0.06)	0.90(±0.03)	0.92(±0.04)	0.91(±0.03)	0.92(±0.04)	2526	256	180	2610
16	0.94(±0.05)	0.90(±0.03)	0.92(±0.03)	0.91(±0.03)	0.92(±0.04)	2528	254	180	2610
17	0.94(±0.03)	0.91(±0.01)	0.93(±0.02)	0.91(±0.02)	0.93(±0.02)	2533	249	153	2637
18	0.94(±0.04)	0.91(±0.02)	0.93(±0.04)	0.91(±0.02)	0.93(±0.03)	2537	245	152	2638
19	0.94(±0.05)	0.91(±0.02)	0.92(±0.03)	0.91(±0.02)	0.92(±0.03)	2520	262	163	2627

Table S7. Performance analysis of GB algorithm using KNN data imputation and Extra Tree feature ranking.

Number of Features	Sensitivity (Recall)	Specificity	Accuracy	Precision	F1_Score	Non-CKD		CKD	
						True Negative	False Positive	False Negative	True Positive
1	0.96(±0.01)	0.66(±0.03)	0.81(±0.01)	0.74(±0.02)	0.84(±0.01)	1824	958	100	2690
2	0.96(±0.01)	0.66(±0.03)	0.81(±0.01)	0.74(±0.02)	0.84(±0.01)	1824	958	100	2690
3	0.92(±0.08)	0.77(±0.04)	0.85(±0.04)	0.80(±0.03)	0.86(±0.04)	2148	634	219	2571
4	0.92(±0.08)	0.84(±0.04)	0.88(±0.03)	0.85(±0.02)	0.88(±0.04)	2331	451	228	2562
5	0.92(±0.09)	0.85(±0.05)	0.89(±0.03)	0.86(±0.03)	0.89(±0.04)	2360	422	216	2574
6	0.92(±0.07)	0.85(±0.03)	0.89(±0.03)	0.86(±0.02)	0.89(±0.03)	2368	414	214	2576
7	0.93(±0.08)	0.85(±0.04)	0.89(±0.03)	0.86(±0.02)	0.89(±0.04)	2370	412	204	2586
8	0.92(±0.09)	0.85(±0.03)	0.89(±0.04)	0.86(±0.02)	0.89(±0.04)	2369	413	219	2571
9	0.93(±0.08)	0.86(±0.02)	0.90(±0.03)	0.87(±0.02)	0.90(±0.04)	2395	387	195	2595
10	0.93(±0.06)	0.86(±0.03)	0.90(±0.03)	0.87(±0.02)	0.90(±0.03)	2391	391	187	2603
11	0.93(±0.05)	0.86(±0.02)	0.90(±0.03)	0.87(±0.02)	0.90(±0.03)	2394	388	183	2607
12	0.93(±0.05)	0.87(±0.03)	0.90(±0.02)	0.88(±0.02)	0.90(±0.02)	2413	369	188	2602
13	0.93(±0.06)	0.87(±0.01)	0.90(±0.03)	0.88(±0.02)	0.90(±0.04)	2426	356	203	2587
14	0.92(±0.06)	0.86(±0.02)	0.89(±0.03)	0.87(±0.02)	0.90(±0.03)	2405	377	212	2578
15	0.92(±0.06)	0.86(±0.02)	0.89(±0.03)	0.87(±0.02)	0.90(±0.03)	2405	377	212	2578
16	0.93(±0.06)	0.87(±0.02)	0.90(±0.03)	0.88(±0.02)	0.90(±0.03)	2425	357	204	2586
17	0.93(±0.05)	0.86(±0.02)	0.90(±0.03)	0.87(±0.02)	0.90(±0.03)	2404	378	201	2589
18	0.93(±0.05)	0.86(±0.02)	0.90(±0.03)	0.87(±0.02)	0.90(±0.03)	2404	378	201	2589
19	0.92(±0.06)	0.86(±0.02)	0.89(±0.04)	0.87(±0.02)	0.90(±0.04)	2406	376	216	2574

Table S8. Performance analysis of RF algorithm using KNN data imputation and XGB feature ranking.

Number of Features	Sensitivity (Recall)	Specificity	Accuracy	Precision	F1_Score	Non-CKD		CKD	
						True Negative	False Positive	False Negative	True Positive
1	0.96(±0.01)	0.66(±0.03)	0.81(±0.01)	0.74(±0.02)	0.84(±0.01)	1824	958	100	2690
2	0.96(±0.01)	0.66(±0.03)	0.81(±0.01)	0.74(±0.02)	0.84(±0.01)	1824	958	100	2690
3	0.95(±0.01)	0.68(±0.04)	0.82(±0.01)	0.75(±0.02)	0.84(±0.01)	1894	888	132	2658
4	0.86(±0.09)	0.90(±0.02)	0.88(±0.04)	0.89(±0.01)	0.88(±0.05)	2500	282	396	2394
5	0.93(±0.11)	0.90(±0.02)	0.92(±0.05)	0.91(±0.02)	0.92(±0.06)	2510	272	190	2600
6	0.96(±0.05)	0.91(±0.01)	0.93(±0.02)	0.91(±0.01)	0.93(±0.03)	2525	257	119	2671
7	0.96(±0.04)	0.91(±0.01)	0.94(±0.01)	0.91(±0.01)	0.94(±0.02)	2531	251	106	2684
8	0.97(±0.03)	0.91(±0.02)	0.94(±0.01)	0.92(±0.02)	0.94(±0.01)	2540	242	94	2696
9	0.97(±0.03)	0.92(±0.02)	0.94(±0.02)	0.92(±0.01)	0.94(±0.02)	2552	230	96	2694
10	0.98(±0.02)	0.93(±0.02)	0.95(±0.01)	0.93(±0.02)	0.95(±0.01)	2579	203	60	2730
11	0.98(±0.01)	0.93(±0.01)	0.96(±0.01)	0.94(±0.01)	0.96(±0.01)	2593	189	59	2731
12	0.98(±0.01)	0.93(±0.02)	0.96(±0.01)	0.94(±0.02)	0.96(±0.01)	2593	189	47	2743
13	0.98(±0.01)	0.93(±0.02)	0.96(±0.01)	0.94(±0.02)	0.96(±0.01)	2595	187	52	2738
14	0.98(±0.01)	0.93(±0.02)	0.96(±0.01)	0.94(±0.02)	0.96(±0.01)	2596	186	49	2741
15	0.99(±0.01)	0.94(±0.01)	0.96(±0.00)	0.94(±0.01)	0.96(±0.00)	2611	171	37	2753
16	0.98(±0.01)	0.94(±0.02)	0.96(±0.01)	0.94(±0.02)	0.96(±0.00)	2613	169	43	2747
17	0.99(±0.01)	0.94(±0.02)	0.96(±0.01)	0.94(±0.02)	0.96(±0.01)	2609	173	37	2753
18	0.99(±0.01)	0.94(±0.02)	0.96(±0.01)	0.94(±0.02)	0.96(±0.01)	2613	169	36	2754
19	0.99(±0.01)	0.93(±0.02)	0.96(±0.01)	0.94(±0.02)	0.96(±0.01)	2599	183	38	2752

Table S9. Performance analysis of XGB algorithm using KNN data imputation and Extra Tree feature ranking.

Number of Features	Sensitivity (Recall)	Specificity	Accuracy	Precision	F1_Score	Non-CKD		CKD	
						True Negative	False Positive	False Negative	True Positive
1	0.96(±0.01)	0.66(±0.03)	0.81(±0.01)	0.74(±0.02)	0.84(±0.01)	1824	958	100	2690
2	0.96(±0.01)	0.66(±0.03)	0.81(±0.01)	0.74(±0.02)	0.84(±0.01)	1824	958	100	2690
3	0.91(±0.22)	0.79(±0.08)	0.85(±0.07)	0.81(±0.03)	0.85(±0.10)	2185	597	250	2540
4	0.93(±0.12)	0.86(±0.05)	0.90(±0.04)	0.87(±0.03)	0.90(±0.05)	2400	382	201	2589
5	0.93(±0.10)	0.86(±0.03)	0.90(±0.04)	0.87(±0.02)	0.90(±0.05)	2405	377	188	2602
6	0.95(±0.08)	0.87(±0.03)	0.91(±0.04)	0.88(±0.02)	0.91(±0.04)	2411	371	147	2643
7	0.95(±0.07)	0.87(±0.02)	0.91(±0.03)	0.88(±0.02)	0.91(±0.04)	2419	363	141	2649
8	0.96(±0.06)	0.86(±0.02)	0.91(±0.02)	0.88(±0.01)	0.91(±0.03)	2404	378	123	2667
9	0.96(±0.04)	0.86(±0.03)	0.91(±0.02)	0.88(±0.02)	0.92(±0.02)	2399	383	107	2683
10	0.97(±0.03)	0.87(±0.02)	0.92(±0.01)	0.88(±0.01)	0.92(±0.01)	2424	358	95	2695
11	0.96(±0.03)	0.87(±0.02)	0.92(±0.02)	0.88(±0.01)	0.92(±0.02)	2409	373	98	2692
12	0.96(±0.02)	0.88(±0.02)	0.92(±0.02)	0.89(±0.01)	0.92(±0.02)	2442	340	108	2682
13	0.96(±0.03)	0.88(±0.01)	0.92(±0.02)	0.89(±0.01)	0.92(±0.02)	2444	338	112	2678
14	0.96(±0.03)	0.88(±0.02)	0.92(±0.02)	0.89(±0.01)	0.92(±0.02)	2453	329	113	2677
15	0.96(±0.03)	0.88(±0.01)	0.92(±0.02)	0.89(±0.01)	0.92(±0.02)	2453	329	119	2671
16	0.96(±0.03)	0.88(±0.01)	0.92(±0.02)	0.89(±0.01)	0.92(±0.02)	2447	335	118	2672
17	0.96(±0.03)	0.88(±0.02)	0.92(±0.02)	0.89(±0.02)	0.92(±0.02)	2436	346	106	2684
18	0.96(±0.03)	0.88(±0.02)	0.92(±0.02)	0.89(±0.02)	0.92(±0.02)	2438	344	106	2684
19	0.96(±0.03)	0.87(±0.02)	0.92(±0.02)	0.88(±0.02)	0.92(±0.02)	2432	350	101	2689

Table S10. Performance analysis of LightGBM algorithm using KNN data imputation and XGB feature ranking.

Number of Features	Sensitivity (Recall)	Specificity	Accuracy	Precision	F1_Score	Non-CKD		CKD	
						True Negative	False Positive	False Negative	True Positive
1	0.96(±0.01)	0.65(±0.03)	0.81(±0.02)	0.74(±0.02)	0.83(±0.01)	1822	961	104	2686
2	0.96(±0.01)	0.65(±0.03)	0.81(±0.02)	0.74(±0.02)	0.83(±0.01)	1822	961	104	2686
3	0.95(±0.01)	0.68(±0.04)	0.82(±0.02)	0.75(±0.02)	0.84(±0.02)	1892	891	138	2652
4	0.91(±0.02)	0.82(±0.04)	0.87(±0.02)	0.83(±0.03)	0.87(±0.02)	2269	514	238	2552
5	0.91(±0.31)	0.93(±0.04)	0.92(±0.14)	0.93(±0.02)	0.91(±0.18)	2577	206	263	2527
6	0.91(±0.28)	0.93(±0.04)	0.92(±0.13)	0.93(±0.03)	0.91(±0.16)	2578	205	247	2543
7	0.90(±0.35)	0.93(±0.06)	0.92(±0.15)	0.94(±0.05)	0.91(±0.20)	2598	185	278	2512
8	0.91(±0.32)	0.94(±0.05)	0.92(±0.14)	0.94(±0.04)	0.91(±0.18)	2609	174	248	2542
9	0.92(±0.28)	0.94(±0.05)	0.93(±0.12)	0.94(±0.04)	0.92(±0.15)	2614	169	212	2578
10	0.93(±0.25)	0.94(±0.04)	0.93(±0.11)	0.94(±0.03)	0.93(±0.13)	2610	173	190	2600
11	0.94(±0.20)	0.94(±0.04)	0.94(±0.08)	0.94(±0.03)	0.94(±0.10)	2615	168	156	2634
12	0.96(±0.16)	0.94(±0.04)	0.95(±0.06)	0.95(±0.03)	0.95(±0.07)	2626	157	119	2671
13	0.95(±0.18)	0.94(±0.04)	0.95(±0.07)	0.94(±0.03)	0.94(±0.08)	2619	164	135	2655
14	0.95(±0.16)	0.94(±0.04)	0.95(±0.07)	0.95(±0.03)	0.95(±0.08)	2625	158	130	2660
15	0.96(±0.16)	0.95(±0.03)	0.95(±0.07)	0.95(±0.03)	0.95(±0.08)	2635	148	122	2668
16	0.96(±0.17)	0.95(±0.04)	0.95(±0.07)	0.95(±0.03)	0.95(±0.08)	2632	151	125	2665
17	0.96(±0.13)	0.94(±0.03)	0.95(±0.05)	0.94(±0.02)	0.95(±0.06)	2619	164	98	2692
18	0.96(±0.13)	0.94(±0.03)	0.95(±0.05)	0.95(±0.02)	0.95(±0.06)	2626	157	101	2689
19	0.96(±0.14)	0.94(±0.04)	0.95(±0.05)	0.94(±0.03)	0.95(±0.06)	2617	166	104	2686
